# Cancer cell metabolic plasticity allows resistance to NAMPT inhibition but invariably induces dependence on LDHA

**DOI:** 10.1186/s40170-018-0174-7

**Published:** 2018-03-08

**Authors:** Natthakan Thongon, Chiara Zucal, Vito Giuseppe D’Agostino, Toma Tebaldi, Silvia Ravera, Federica Zamporlini, Francesco Piacente, Ruxanda Moschoi, Nadia Raffaelli, Alessandro Quattrone, Alessio Nencioni, Jean-Francois Peyron, Alessandro Provenzani

**Affiliations:** 1 Center For Integrative Biology (CIBIO), University of Trento, via Sommarive 9, Trento, Italy; 2 Department of Pharmacy, Biochemistry Laboratory, University of Genova, Genova, Italy; 3 Department of Agricultural, Food and Environmental Sciences, Polytechnic University of Marche, Ancona, Italy; 4 Department of Internal Medicine, University of Genoa, Genoa, Italy; 5 Université Côte d’Azur, Centre Méditerranéen de Médecine Moléculaire (C3M), INSERM U1065, Nice, France

**Keywords:** NAMPT, Amino acid metabolism, Drug resistance, LDHA, QPRT

## Abstract

**Background:**

Inhibitors of nicotinamide phosphoribosyltransferase (NAMPT), the rate-limiting enzyme in NAD^+^ biosynthesis from nicotinamide, exhibit anticancer effects in preclinical models. However, continuous exposure to NAMPT inhibitors, such as FK866, can induce acquired resistance.

**Methods:**

We developed FK866-resistant CCRF-CEM (T cell acute lymphoblastic leukemia) and MDA MB231 (breast cancer) models, and by exploiting an integrated approach based on genetic, biochemical, and genome wide analyses, we annotated the drug resistance mechanisms.

**Results:**

Acquired resistance to FK866 was independent of NAMPT mutations but rather was based on a shift towards a glycolytic metabolism and on lactate dehydrogenase A (LDHA) activity. In addition, resistant CCRF-CEM cells, which exhibit high quinolinate phosphoribosyltransferase (QPRT) activity, also exploited amino acid catabolism as an alternative source for NAD^+^ production, becoming addicted to tryptophan and glutamine and sensitive to treatment with the amino acid transport inhibitor JPH203 and with l-asparaginase, which affects glutamine exploitation. Vice versa, in line with their low QPRT expression, FK866-resistant MDA MB231 did not rely on amino acids for their resistance phenotype.

**Conclusions:**

Our study identifies novel mechanisms of resistance to NAMPT inhibition, which may be useful to design more rational strategies for targeting cancer metabolism.

**Electronic supplementary material:**

The online version of this article (10.1186/s40170-018-0174-7) contains supplementary material, which is available to authorized users.

## Background

The development of drug resistance during chemo- or targeted-therapies is a common event, which typically underlies disease relapse and is frequently associated with a poor prognosis. Drug resistance is usually classified as intrinsic (also known as primary) or acquired (also known as secondary), depending on whether it is already present in cancer cells at treatment onset or, rather, it eventually develops during treatment [1]. The molecular mechanisms responsible for drug resistance vary depending on the agent used, but some common mechanisms frequently do come into play [2]. These include activation of drug efflux pumps [3], mutations of the targeted protein [4, 5], activation of compensation/buffer mechanisms [6], and evasion of apoptosis [7, 8].

Nicotinamide phosphoribosyltransferase (NAMPT) is a key enzyme for NAD^+^ production from nicotinamide, and NAMPT expression by a tumor predicts a poor prognosis [9] in colon cancer [10], gastric cancer [11], and lymphoma [12]. Therefore, NAMPT has been proposed as a viable cancer drug target, and many molecular agents have been developed that inhibit its enzymatic function, such as FK866, GNE618, and CHS-828 [13, 14]. Clinical trials showed a good tolerability for these drugs, with thrombocytopenia being the most common dose-limiting adverse event. Regrettably, no objective tumor remission in response to these NAMPT inhibitors was observed [15]. New NAMPT inhibitors are under development which will hopefully overcome these limitations [16]. In addition, given the unique mode of action of NAMPT inhibitors, their association with other types of anticancer agents, such as chemotherapeutics, ibrutinib, bortezomib, tumor necrosis factor-related apoptosis-inducing ligand (TRAIL), and cyclosporine-A, has been proposed as a way to boost the efficacy of single treatments [17–22].

Yet, the key issue that limits the benefit of NAMPT inhibitors remains our limited understanding of the factors that determine cancer cell response to these agents vs. resistance across different cancer types [23]. Several NAD^+^ biosynthetic pathways that are alternative to that controlled by NAMPT exist. The Preiss-Handler pathway for NAD^+^ production utilizes nicotinic acid (NA) and requires the expression of nicotinic acid phosphoribosyltransferase (NAPRT) for the first enzymatic step to convert NA to nicotinic acid mononucleotide (NAMN) [19]. NAMPT inhibition is specifically effective in cancer cells in which NAPRT-mediated NAD^+^ production is not functioning [24]. Treatment with NAMPT inhibitors combined with NA supplementation was proposed to allow the killing of NAPRT-deficient cancer cells while simultaneously protecting normal cells thanks to the ability of the latter, but not of the former, to exploit NA as NAD^+^ precursor [25]. In addition, NAPRT silencing in a BRCA2-deficient cancer cell line exacerbated DNA damage in response to chemotherapeutics [26]. Acquired resistance to NAMPT inhibitors was also reported and was proposed to be mostly due to mutations in NAMPT itself, as in the case of HCT-116 colon cancer cells, which were induced to become resistant to CHS-828. A resistant clone exhibiting acquired resistance to CHS-828 was obtained by continuous cell exposure to increasing sub-lethal concentrations of the drug. Sequencing of the *NAMPT* gene in the CHS-828-resistant cell line revealed a single point mutation of amino acid 217 from glycine to arginine (G217R) [27]. Similarly, other mutations in the *NAMPT* gene (mapped to H191R, to D93del, and to Q388R) were also reported in resistant cell lines developed from HCT-116 and from NYH human small cell lung carcinoma cells and were found to confer drug resistance to other NAMPT inhibitors, such as FK866 and CHS-828. All of these mutations were found to structurally modify the inhibitor-binding pocket (G217R and H191R), preventing drug efficacy. The resistant cell lines showed tumorigenicity in mouse xenografts and in vivo resistance to NAMPT inhibitors [13]. Six mutations in the *NAMPT* gene were reported in GNE-618-resistant cells derived from rhabdosarcoma (RD), pancreatic cancer (MiaPaCa-2), and non-small cell lung cancer (NCI-H460) cells [28]. In addition to the previously identified G217R and D93del mutations, the new G217A, G217V, S165F, and S165Y mutations were reported as resistance-causing mutations in this study. Resistant cell lines were observed to be 10- to 1000-fold less sensitive to the NAMPT inhibitor GNE-618 as compared to the parental cells. Tumor xenografts established from NCI-H460 cells expressing the S165Y mutation were resistant to high doses of GNE-618 [28]. Notably, no cross-resistance to CHS-828 and FK866 was detected, suggesting that resistance to NAMPT inhibitors could be molecule-specific. Therefore, to date, tumor cell resistance to NAMPT inhibitors has been mostly ascribed to point mutations that are either proximal or distal to the enzyme substrate binding sites with the notable exception of one case of NAMPT inhibitor resistance, that was related to increased activity of the enzyme quinolinate phosphoribosyltransferase (QPRT), which mediates NAD^+^ production from tryptophan [29].

**Fig. 1:**
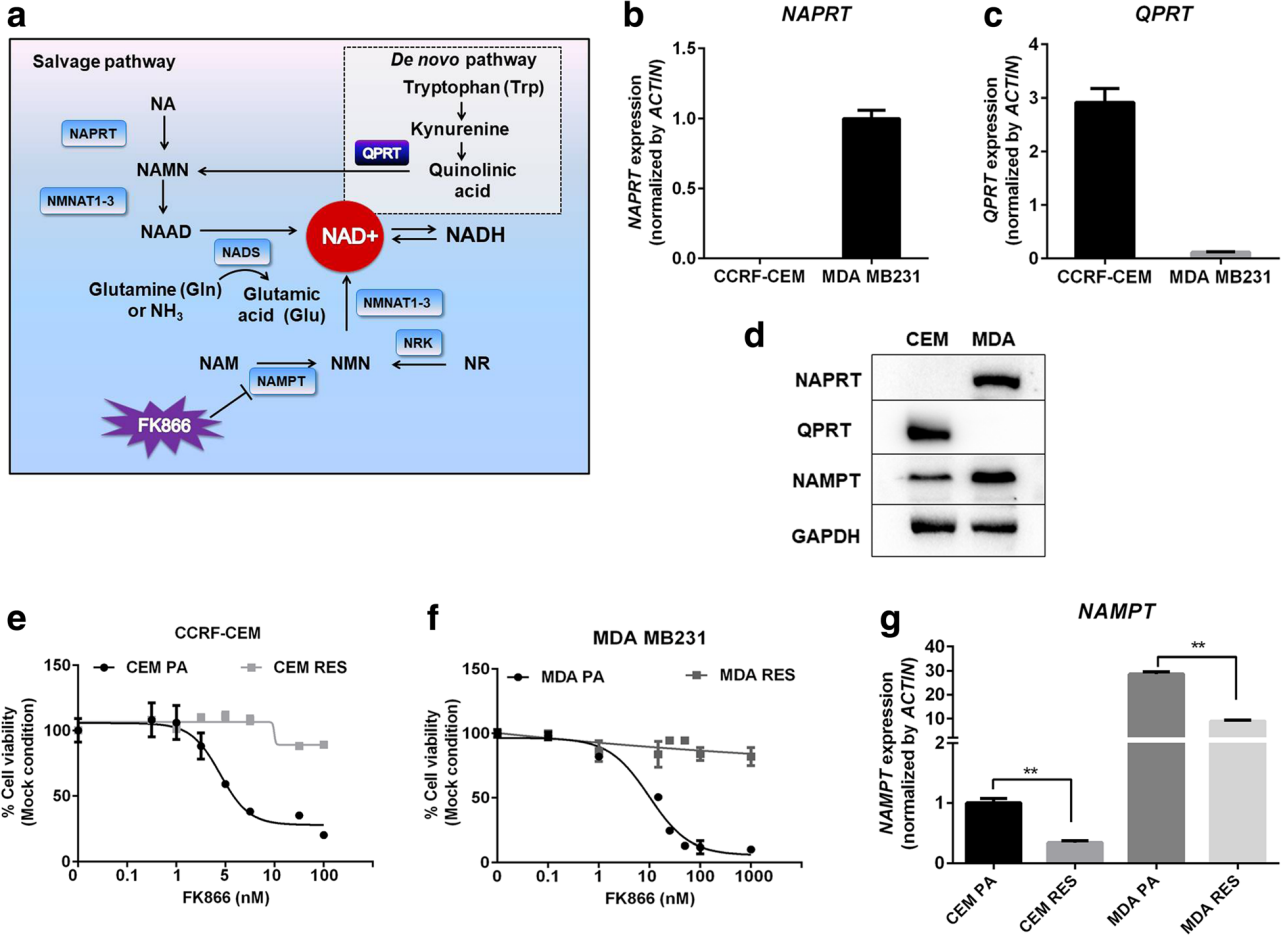
Overview of NAD biosynthesis pathway and genetic differences in NAD(H) biosynthesis pathway between CCRF-CEM and MDA MB231 cell lines. **a** Schematic representation of salvage and de novo pathway of NAD biosynthesis. Pharmacological target of FK866 inhibiting NAMPT is displayed. Abbreviations: NAD, nicotinamide adenine dinucleotide; NA, nicotinic acid; NAMN, nicotinic acid mononucleotide; NAAD, nicotinic acid adenine dinucleotide; NAPRT, nicotinic acid phosphoribosyltransferase; NMNAT, nicotinamide mononucleotide adenylyltransferase; NADS, NAD synthase; NAM, nicotinamide; NMN, nicotinamide mononucleotide; NAMPT, nicotinamide phosphoribosyltransferase; NR, nicotinamide riboside; NRK, nicotinamide riboside kinase; QPRT, quinolinate phosphoribosyltransferase. **b**, **c** Expression of *NAPRT* and *QPRT* were determined in CCRF-CEM (CEM) and MDA MB231 (MDA) cell lines. **d** Western blot showing endogenous level of NAPRT, QPRT, and NAMPT in CEM and MDA cell lines. GAPDH was used as a loading control. **e**, **f** Sensitivity of CEM and MDA cells to FK866. CEM parental (CEM PA) and FK866-resistant CEM (CEM RES) cells (**e**) and MDA parental (MDA PA) and FK866-MDA resistant (MDA RES) cells (**f**) were exposed to various concentrations of FK866 (0.1–100 nM) for 48 h. Percentage of cell viability and EC50 were analyzed by MTT assay. **g** Downregulation of *NAMPT* in the resistant cells. Quantitative RT-PCR showing the expression of *NAMPT* in CEM PA, CEM RES, MDA PA, and MDA RES cells. *ACTIN* was used as a housekeeping gene

One strategy to overcome drug resistance is to identify and target the specific vulnerabilities or addictions that arise in the cancer population. Here, we investigated the molecular mechanism leading to acquired FK866 resistance in two genetically different cancer cell lines, CCRF-CEM T-ALL cells, which are NAPRT-deficient, and MDA MB231 triple-negative breast cancer cells, which, vice versa, express an active NAPRT enzyme but lack QPRT (Table 1). Both cancer cell lines developed resistance to FK866, and interestingly, in both cases, acquired resistance was independent of mutations in *NAMPT* but rather relied on the activation of alternative metabolic pathways that compensated for low NAD(H) level (Fig. 1a). Interestingly, the compensatory mechanisms mediating resistance were found to be profoundly different in the two cell lines, inducing different types of vulnerabilities in the resistant cells and suggesting diversified pharmacological approaches to treat them. However, both types of resistant cells did exhibit a marked reliance on LDHA activity, thus pointing to this enzyme as a crucial crossroad for cancer cell survival and as a new anticancer target.

**Table 1:** Genetic differences between CCRF CEM and MDA MB231 cell lines

Gene expression	Peripheral blood (acute lymphoblastic)	Breast (adenocarcinoma)
	CCRF CEM	MDA MB 231
*NAMPT*	Intermediate	High
*NAPRT*	–	High
*QPRT*	High	Very low
*NRK*	Low	Intermediate
*LDH*	Intermediate	Intermediate
PTEN*	Mutation	–
KRAS*	Mutation	Mutation
TP53*	–	Mutation
BRAF*	–	Mutation
CDKN2A*	–	Mutation

*Source: ATCC cell lines by gene mutation

## Methods

### Cell culture and development of NAMPT-resistant cell models

Human acute lymphoblastic leukemia cells (CCRF-CEM) and human breast cancer cells (MDA MB231) were obtained from the Interlab Cell line Collection bank (ICLC HTL01002) and American Type Culture Collection (ATCC), respectively. CCRF-CEM (CEM) cells were maintained in RPMI 1640 (Invitrogen, Carlsbad, CA), and MDA MB231 (MDA) cells were maintained in DMEM supplemented with 10% fetal bovine serum (FBS, Lonza), 2 mM l-glutamine, and 100 U/ml penicillin-streptomycin (Lonza). They were cultured at 37 °C, 5% CO_2_ incubator. NAMPT-resistant cell models were gradually developed by increasing the concentration of FK866 (sc-205325, Santa Cruz) up to 500 nM in both cell lines. Cell viability was determined by exclusion of 0.2% trypan blue. The FK866-resistant sublines, CEM RES and MDA RES, were maintained in the presence of 100 nM FK866 in the culture medium. A final 100-nM FK866-resistant subline (CEM RES) was used in all experiments.

### In vitro cell viability and drug combination assays

CCRF-CEM and MDA MB231 cells were treated with FK866, CHS-828 (200484-11-3, Cayman Chemical), oligomycin A (75351, Sigma), 2-deoxyglucose (D8375, Sigma), GSK2837808A (GSK) (5189, Tocris), JPH203 (a selective L-type amino acid transporter), and l-asparaginase (11185, Sigma) for 48 h. JPH203 was kindly obtained from Dr. Peyron [30]. In vitro drug sensitivity was assessed by the colorimetric methyl-thiazolyltetrazolium (MTT) assay (sigma), XTT proliferation kit (sigma), and OZBlue Cell Viability kit (OZbiosciences). Combination treatment of FK866 with JPH203 was performed and 48 h after combination treatment, Cell viability was determined using XTT assay (Invitrogen) and DAPI staining. Percentage of cell death was subjected for drug combination analysis as described by combination index (CI). CI was analyzed using CompuSyn software V1.0 by the method of Chou and Talalay [31]. CI < 1 indicates drug synergistic effect, CI > 1 indicates drug antagonistic effect, and CI = 1 indicates drug additive effect.

### Determination of NAD(H), ATP levels, and rate of protein translation

Cells were treated with FK866 for 48 h. Intracellular NAD(H) and ATP content were assessed with a NAD/NADH-Glo (G9071, Promega) and CellTiter-Glo Luminescent Cell Viability Assay (G7571, Promega) according to the manufacturer’s protocol. Fluorescence data were measured and normalized to protein concentration in the cell lysates (Bradford Reagent, Sigma). Rate of protein translation was analyzed by Click-iT AHA Alexa Fluor 488 Protein Synthesis Assay (Life Technologies) according to the manufacturer’s protocol. Cells were treated with FK866 for 45 h and further incubated for 3H with 50 μM AHA in l-methionine-free medium (RPMI-1640 Medium, Sigma-Aldrich) containing the drug (or DMSO). After fixation and permeabilization, AHA incorporation was assessed by flow cytometry. 7-AAD Staining Solution (0.25 μg/sample) allowed the exclusion of non-viable cells. Flow cytometry experiments were carried out on two biological replicates, and statistics were based on acquisition of 50,000 events/sample.

For the HPLC analysis of NAD and NMN (nicotinamide mononucleotide), nucleotides were extracted by resuspending cell pellets in 0.4 M HClO_4_. After 5 min on ice, samples were centrifuged at 16,000×*g* for 5 min. Pellets were resuspended in 100 μl formic acid, incubated 5 min at 37 °C, and used for the determination of the protein concentration (Bradford reagent). Supernatants were neutralized with 1 M K_2_CO_3_ and used for NAD and NMN determination. NAD was quantified by UV C18-HPLC under ion-pairing conditions [32]. NMN was first derivatized with acetophenone as described in [33] and then quantified by spectrofluorometric HPLC analysis as described in [32]. Values of NAD and NMN levels were referred to the protein concentration.

### Western blot analysis

Amino acid depletion experiment was conducted in 20:80 mixture of normal RPMI-1640 (10% FBS + 2 mM l-glutamine) with Eagle’s balanced salt solution (E3024; sigma) supplemented with 10% FBS. Cells were treated with FK866, JPH203, L-Asp, and indicated combinations for 24 h in the present or absent of amino acid supplementation including 2 mM l-glutamine (Sigma), 0.25 mM l-tryptophan (Sigma), 1× essential amino acid mixture (EAA) (Sigma), and 1× non-essential amino acid (NEAA) (Sigma). Samples were lysed in RIPA lysis buffer supplemented with Protease Inhibitor Cocktail (Thermo Scientific). Equal amounts of proteins were separated by SDS–PAGE. The antibodies used were the following: QPRT (TA501520) from OriGene; 4EBP1 (sc-6936), p-4EBP1(Ser 65/Thr 70; sc-12884), EIF4E (sc-9976), p-EIF4E (Ser209; sc-12885), MTOR (sc-8319), NAMPT(sc-130058), GAPDH (sc-32233), and LDHA (sc-137243) from Santa Cruz; NAPRT (ab-211529), EIF2A (ab26197), p-EIF2A (Ser 51; ab32157), and p-MTOR (Ser 2448; ab1093) from Abcam; and AMPKα (2603), p-AMPKα (Thr 172; 2531), ACTIN (3700), and CHOP (2895) from Cell Signaling. ACTIN and GAPDH were used as protein loading control. Densitometric analysis and normalization relative to control was conducted using ImageJ software. A representative western blot of three-independent biological experiments is shown.

### shRNA and siRNA transfection

Lentiviral vector particles (LVPs) were generated in Lenti-HEK293 cells. Briefly, cells were co-transfected with a Δ891 and a VSV-G encoding vector along with a shRNA transfer lentiviral plasmid PLKO vector and a shNAPRT plasmid (a gift of Dr. Alessio Nencioni). Seventy-two hours post-transfection, viral supernatants were collected, filtrated, and performed quantification. MDA cells were stably transduced with shNAPRT, and selection of puromycin (2.5 μg/ml) was performed. Downregulation of LDHA in MDA cells was performed by transient transfection of siRNA of *LDHA* using INTERFERin in vitro siRNA/miRNA transfection reagent (Polyplus) for 48 h. Then, cells were treated with FK866 for 48 h.

### RNA extraction and real-time PCR

Total RNA was extracted using RNA extraction kit (ZYMO research), and contaminant DNA was removed by RNase-Free DNase kit (ZYMO research). Synthesis of cDNA was carried out on RevertAid RT kit (K1691, Themoscientific). Real-time qPCR analyses were performed with triplicates using KAPA SYBR FAST Universal qPCR Kit on a CFX96 real-time PCR Detection system (Bio-Rad). Expression levels are always given relative to *ACTIN*, *18S*, or *GAPDH*. Primer’s sequences are provided in Additional file 1: Table S2.

### Analysis of mitochondria functions and cellular compartment ATP production

Cells were costained with 100 nM MitoTracker^TM^ CMTMRos Orange (Invitrogen) and DRAQ5 Fluorescent Probe Solution (62251) (Thermo Scientific^TM^) for 30 min. Mitochondria images, number, and intensity were acquired by PerkinElmer Operetta and analyzed by harmony analysis software. Loss functional mitochondria was monitored by the sensitivity to carbonilcyanide p-triflouromethoxyphenylhydrazone (FCCP).

### Mitochondria and cytosolic ATP production

Cells were treated in triplicate with control (PBS), oligomycin A (10 μg/ml; Sigma), sodium iodoacetate (100 μM; Sigma), or both. Following a 1-h incubation at 37 °C, ATP levels were measured using the CellTiter-Glo Luminescent Cell Viability Assay (G7571, Promega) according to the manufacturer’s protocol. Luminescence was then recorded on a Berthold Technologies Luminoscan. Oligomycin A is used to block ATP production by oxidative phosphorylation, and iodoacetate prevents the ATP production through glycolysis. The addition of both molecules is used to determine residual ATP levels which are subtracted for each condition.

### Oxygen consumption measurements

Oxygen consumption was measured at 37 °C in a closed chamber, using an amperometric electrode (Unisense Microrespiration, Unisense A/S, Denmark). Two-hundred thousand cells were permeabilized with 0.03 mg/ml digitonin for 1 min, centrifuged for 9 min at 1000 rpm, and resuspended in a buffer containing 137 mM NaCl, 5 mM KCl, 0.7 mM KH2PO4, 25 mM Tris–HCl, pH 7.4, and 25 mg/ml ampicillin. The same solution was used in the oxymetric measurements; 10 mM pyruvate plus 5 mM malate were added to stimulate the pathway composed by complexes I, III, and IV. Then, 20 mM succinate was added to stimulate the pathway formed by complexes II, III, and IV. To observe the ADP-stimulated respiration rates, 0.08 mM ADP was added after pyruvate and malate or succinate addition. To verify whether oxygen consumption is really due to the electron transport chain 0.1 mM rotenone or 50 μM antimycin A, specific inhibitors of complex I or complex III were used (data not shown). The respiratory rates were expressed as nmol O/min/10^6^ cells [34].

### ATP synthase activity assay

To evaluate the ATP synthase activity, 100,000 cells were incubated for 10 min at 37 °C in a medium containing 10 mM Tris–HCl, pH 7.4, 100 mM KCl, 5 mM KH_2_PO_4_, 1 mM EGTA, 2.5 mM EDTA, 5 mM MgCl_2_, 0.6 mM ouabain, and 25 mg/ml ampicillin. Afterward, ATP synthesis was induced by the addition of 10 mM pyruvate plus 5 mM malate or 20 mM succinate to stimulate the pathways composed by complexes I, III, and IV or complexes II, III, and IV, respectively. The reaction was monitored for 2 min, every 30 s, in a luminometer (GloMax® 20/20n Luminometer, Promega Italia, Milano, Italy), by the luciferin/luciferase chemiluminescent method, with ATP standard solutions between 10–8 and 10–5 M (luciferin/luciferase ATP bioluminescence assay kit CLSII, Roche, Basel, Switzerland). Data were expressed as nmol ATP produced/min/10^6^ cells [35]. The oxidative phosphorylation efficiency (P/O ratio) was calculated as the ratio between the concentration of the produced ATP and the amount of consumed oxygen in the presence of respiring substrate and ADP. When the oxygen consumption is completed devoted to the energy production, the P/O ratio should be around 2.5 and 1.5 after pyruvate + malate or succinate addition, respectively [36].

### Glucose consumption

Glucose consumption was evaluated by the hexokinase (HK) and glucose-6-phosphate dehydrogenase (G6PD) coupling system, following the reduction of NADP at 340 nm. The assay medium contained 100 mM Tris–HCl, pH 7.4, 2 mM ATP, 10 mM NADP, 2 mM MgCl2, 2 IU of hexokinase, and 2 IU of glucose-6-phosphate dehydrogenase. Data was normalized to the cell number and expressed as mM glucose consumed/10^6^ cells [37].

### Lactate release assay

Lactate concentration was assayed spectrophotometrically in the growth medium, following the reduction of NAD^+^ at 340 nm. The assay medium contained 100 mM Tris–HCl (pH 8), 5 mM NAD^+^, and 1 IU/ml of lactate dehydrogenase. Samples were analyzed before and after the addition of 4 μg of purified lactate dehydrogenase. Data was normalized to the cell number and expressed as mM lactate released/10^6^ cells [37].

### Glycolytic enzyme assays

All enzymatic assays were performed on 50 μg of total protein. The reaction mixtures used for the determination of each enzyme activity were prepared as follows [38]: hexokinase (HK, EC 2.7.1.1): 100 mM Tris–HCl, pH 7.4, 5 mM MgCl2, 200 mM glucose, 1 mM ATP, 0.91 mM NADP, and 0.55 IU/ml of glucose-6-phosphate dehydrogenase (G6PD); phosphofructokinase (PFK, EC 2.7.1.11): 100 mM Tris–HCl, pH 7.4, 2 mM MgCl2, 5 mM KCl, 2 mM fructose 6 phosphate, 1 mM ATP, 0.5 mM phosphoenolpyruvate (PEP), 40 μM rotenone, 0.2 mM NADH, and 2 IU/ml of pyruvate kinase plus lactate dehydrogenase; pyruvate kinase (PK, EC 2.7.1.40): 100 mM Tris–HCl, pH 7.6, 2.5 mM MgCl2, 10 mM KCl, 0.6 mM phosphoenolpyruvate, 40 μM rotenone, 0.2 mM NADH, 5 mM ADP, and 1 IU/ml of lactate dehydrogenase; and lactate dehydrogenase (LDH, EC 1.1.1.27): 100 mM Tris–HCl, pH 9, 5 mM pyruvate, 40 μM rotenone, and 0.2 mM NADH. Each enzymatic activity was expressed as mU/mg of total protein (micromoles/min/mg of protein).

### Activity assay of NAD(H) biosynthetic enzymes

Pellets of about 20 × 10^6^ cultured cells were homogenized with 0.25 ml buffer consisting of 50 mM Tris–HCl, pH 7.5, 0.15 M NaCl, 1 mM dithiothreitol (DTT), 1 mM phenylmethanesulfonyl fluoride, and 0.002 mg/ml each of leupeptin, antipain, chymostatin, and pepstatin. Homogenates were centrifuged at 20,000×*g* for 10 min at 4 °C, and the supernatants were used for the simultaneous assay of QAPRT, NAPRT, NAMPT, NRK, and NADS activities. NMNAT activity was assayed in the homogenates. The activities were measured by the coupled fluorometric assay [39]. Briefly, the products of the enzymes’ catalyzed reactions, i.e., NMN, NAMN, and NAAD, were stoichiometrically converted to NAD^+^ by using ancillary bacterial enzymes, and NAD^+^ was quantified by a fluorometric cycling assay for QAPRT, NAPRT, NRK, and NAMPT activity determination [39]. NADS assay mixture contained 50 mM HEPES/KOH buffer, pH 7.5, 0.1 M KCl, 5 mM MgCl2, 10 mM KF, 4 mM ATP, 20 mM glutamine, 1 mM NAAD, and about 0.15 mg/ml cell lysate. A control mixture without NAAD was processed in parallel. NMNAT assay mixture consisted of 40 mM HEPES/KOH buffer, pH 7.5, 25 mM MgCl2, 10 mM KF, 0.06 mg/ml BSA, 1 mM DTT, 1 mM ATP, 1 mM NMN, and about 0.05 mg/ml cell homogenate. A control mixture without NMN was processed in parallel. Assay mixtures were incubated and processed for the quantitation of the formed NAD(H) as described in [39]. The enzymes’ activity was referred to the protein concentration.

### Flow cytometric cell cycle analysis

CCRF-CEM cells were treated with 10 and 20 μM GSK for 48 h, and they were incubated with the DNA-staining chemical propidium iodide (PI) (FITC/annexin V apoptosis detection kit; BD Pharmingen) for DNA content-based assessment of cell cycle distribution for 10 min. Cell cycle analysis was performed using flow cytometric. Twenty-four hours of cell starvation and 100 ng/ml Nocodazole were used as positive controls. Percentage of G1 phase, S phase, and G2 phase were analyzed using FACS.

### Microarrays

Total RNA extracted from CEM RES cells was hybridized in quadruplicate on the Agilent Human GE 4x44K v2 Microarray (G2519F-026652) and compared with total RNA from parental cells. After quantile normalization and removal of low signals, differentially expressed genes were selected according to a double threshold on the log2 fold change (absolute value > 0.75) and moderated *t* test *p* value (< 0.05) calculated with the Limma package. Functional annotation enrichment analysis of the resulting lists of DEGs was performed with the clusterProfiler Bioconductor package. The significance of over-representation was determined using a *p* value threshold of 0.05. Gene ontology terms with less than 500 associated genes were used for the analysis. Microarray raw data are available on Gene Expression Omnibus under the following accession number: GSE96636.

### Statistical analysis

Values are expressed as mean ± S.D. of three independent biological experiments conducted in technical triplicates. Student’s *t* test or one-way ANOVA was employed to determined statistical significance between control and test groups. Values of *p < 0.05, **p < 0.01, and ***p < 0.001 were considered significant. Data were plotted by Graph prism 6 software.

## Results

### Generation of FK866-resistant cancer cell models

The absence of NAPRT in CCRF-CEM (CEM) cells and the low expression of QPRT in MDA MB231 (MDA) cells were found to be the main differences between the two cell lines with respect to their NAD^+^ production apparatus (Fig. 1b–d). We developed FK866-resistant CEM and MDA cells by using a stepwise increase in FK866 concentration [8, 40] in both cases (Fig. 1e, f). FK866 concentration was increased over a 3-month period, and cells became increasingly resistant according to the dose used, suggesting the occurrence of a quick, clinically relevant, adaptation mechanism (Additional file 2: Figure S1A) [40]. The daughter resistant cells were then compared to the parental FK866-sensitive cells, assessing cell viability by MTT-based assays and by counting viable cells. FK866-resistant cells were kept under continuous exposure to FK866 during routine passages. The calculated EC_50_ after 48 h of treatment with FK866 was 9.8 μM for the resistant CCRF-CEM cells (CEM RES), whereas an EC_50_ of 4.4 nM could be estimated for the parental CEM (CEM PA) (about 2000-fold lower) (Fig. 1e, Additional file 2: Figure S1B). Moreover, CEM RES were still able to proliferate during a long-term (5-day) treatment with 500 nM FK866 (Additional file 2: Figure S1C). FK866-resistant MDA MB231 (MDA RES) cells were completely insensitive to FK866 (an EC_50_ could not be calculated), whereas parental cells showed an EC_50_ of 14.8 nM (Fig. 1f). *NAMPT* mRNA levels were higher in MDA cells as compared to CEM cells, and *NAMPT* expression significantly decreased in both resistant CEM and MDA (Fig. 1g). In the subsequent experiments, we aimed at identifying common mechanisms of resistance to NAMPT inhibition that could pinpoint novel therapeutic strategies.

### Resistant CEM cells have lower NAD(H) levels than parental cells but do not show mutations in *NAMPT* coding sequence

**Fig. 2:**
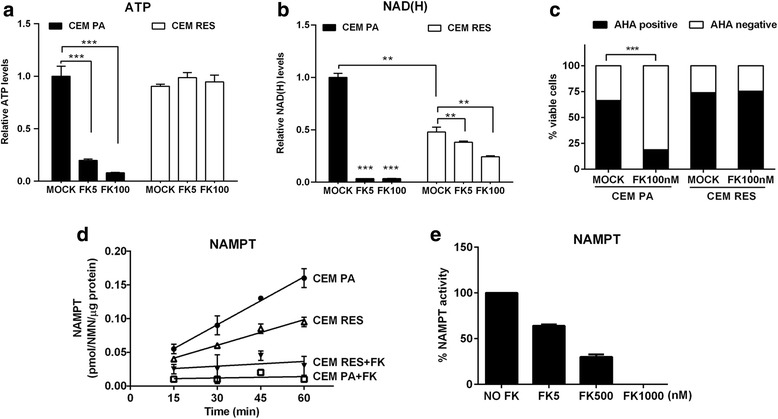
Characterization of FK866-resistant cells. **a**, **b** Administration of FK866 decreases intracellular ATP and NAD(H) levels in CEM PA cells. CEM PA and CEM RES cells were treated with 5 and 100 nM FK866 for 48 h. Relative ATP and NAD(H) levels were normalized to number of viable cells. **c** FK866 inhibits global protein translation. CEM PA and CEM RES cells were treated with 100 nM FK866 for 48 h. Protein synthesis was monitored by Click-it chemistry based on the incorporation of an amino acid analog (AHA). The histogram quantifies FK866-induced protein synthesis arrest in the viable cell population. **d** Time course of the NAMPT enzymatic activity assayed in lysates from CEM PA and CEM RES cells. Assays were performed in the absence and in the presence of 1 μM FK866 in the reaction mixtures. **e** NAMPT enzymatic activity was analyzed in lysates from CEM RES cells in the absence and in the presence of the indicated FK866 concentrations. All data were conducted in three independent experiments with technical triplicates (*p < 0.05, **p < 0.01, ***p < 0.001 compared to Mock)

Acquired resistance to FK866 was not specific for this compound. Instead, it was also observed in response to treatment with CHS-828, a second NAMPT inhibitor (Additional file 2: Figure S1D, E). In both FK866-resistant (CEM RES) and parental CEM (CEM PA), intracellular NAD(H) and ATP levels were measured after a 48-h treatment with FK866 to assess the effect of the drug. While in parental cells, FK866 strongly blunted intracellular ATP stores, in FK866-resistant CCRF-CEM, it had no effect on the nucleotide’s levels (Fig. 2a and Additional file 2: Figure S1F). Interestingly, at baseline, NAD(H) levels in CEM RES were approximately 50% lower than those of untreated parental cells, likely due to the decreased expression of NAMPT. FK866 reduced intracellular NAD(H) amounts in both parental and CEM RES. However, the effect size was much bigger in CEM PA cells than in FK866-resistant cells (Fig. 2b, Additional file 2: Figure S1G). Similar to NAD(H), starting levels of NMN, the product of the NAMPT-catalyzed reaction from NAM, were also lower in CEM RES than in CEM PA cells. NMN decreased upon exposure to FK866 in both parental and resistant cells, even though the decrease was more pronounced in parental than in resistant cells (Additional file 2: Figure S1H). Incorporation of a modified alanine that can be fluorescently labeled (AHA) into nascent proteins after FK866 treatment revealed a decrease in protein synthesis in CEM PA, but not in resistant cells cultivated in the presence of 100 nM FK866 in the medium (Fig. 2c). Cell cycle analysis reported a small, but significant, increase in cycling cells in CEM RES (Additional file 2: Figure S1I).

To define the mechanism underlying acquired FK866 resistance in CEM cells, we first assessed a possible role of multi-drug resistance (MDR). However, treatment with inhibitors of drug efflux pumps, such as verapamil and cyclosporine A, failed to affect cell viability in FK866-resistant CEM exposed to FK866, thus excluding a role for MDR mechanisms in the acquired resistance to FK866 in these cells (Additional file 2: Figure S1J). We subsequently sequenced the *NAMPT* gene in both parental and resistant CEM cells. However, we did not observe any mutation in *NAMPT* coding region in both resistant and parental CEM [13]. By assaying the NAMPT enzymatic activity in the lysates from both cells, we found that it was downregulated by about 50% in the resistant model, in keeping with the lower expression of the protein (Figs. 1g and 2d). Notably, we did observe that NAMPT enzymatic activity was fully inhibited by 1 μM FK866 both in parental and resistant cells (Fig. 2d). Analysis of the dose-response sensitivity in the resistant model showed a 30 and 64% inhibition of the enzyme in the presence of 5 and 500 nM FK866, respectively (Fig. 2e). A 2-month FK866 washout of CEM RES only marginally affected their drug resistance (Additional file 2: Figure S2A, B), suggesting a stable genetic or epigenetic reprogramming as a mechanism underlying NAMPT inhibitor resistance.

### CEM RES cells show constitutive alterations in metabolic pathways

**Fig. 3:**
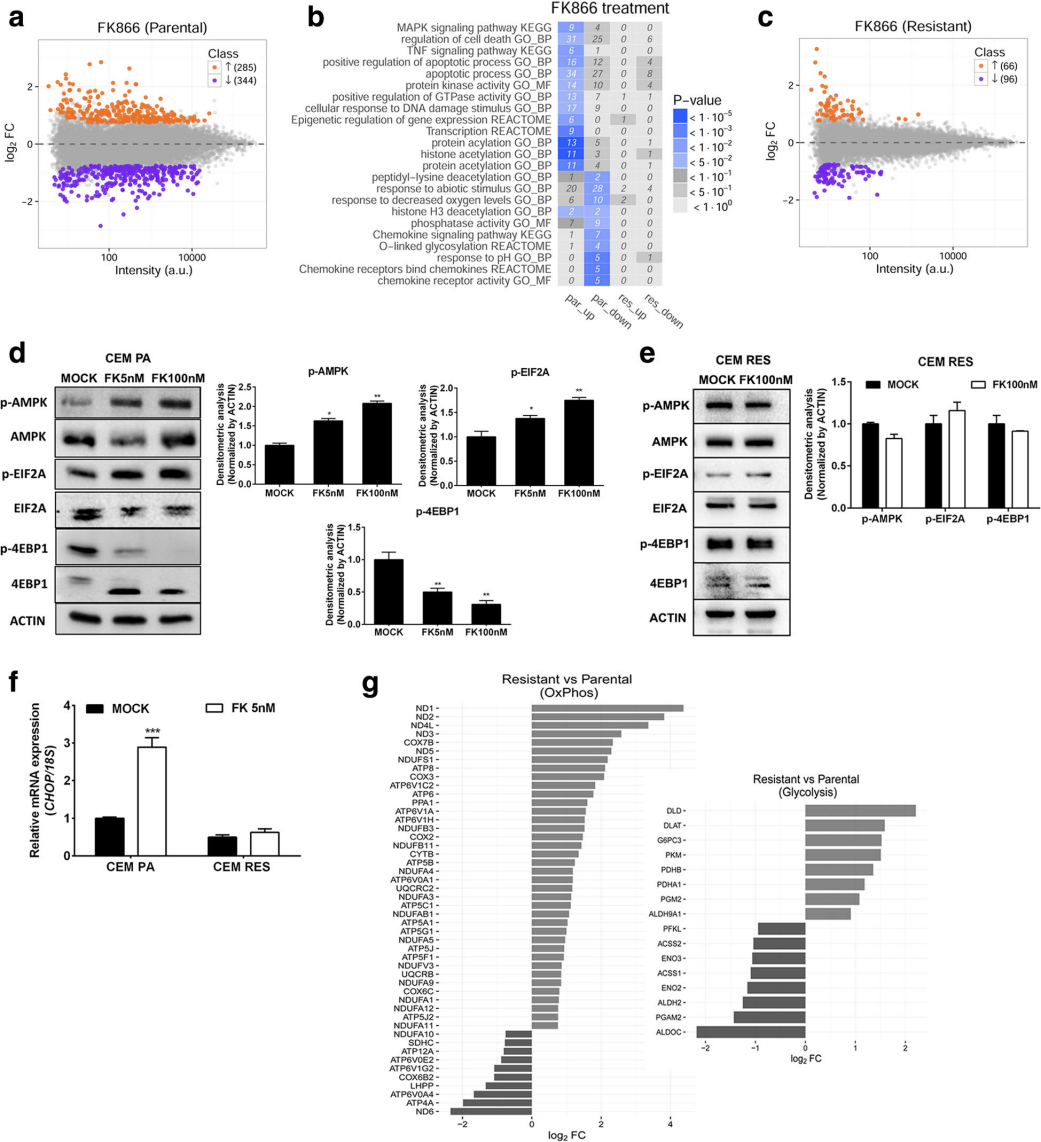
Genome-wide analysis of gene expression in CCRF-CEM cells. **a** MA plot of transcriptome profiling in FK866-treated vs. untreated CEM PA cells. For each gene, the average log_10_ signal against the log_2_ fold change is plotted. Genes significantly up (orange)- or down (violet)-regulated upon FK866 treatment are highlighted. **b** Top enriched gene ontology (GO) terms and KEGG pathways among up- and downregulated genes upon FK866 treatment in CEM PA and CEM RES cells. The heatmap, colored according to enrichment *p* values, displays categories associated with lysine acetylation, modulation of key epigenetic regulators, and transcription factors involved in energetic stress response. The number of genes corresponding to each term is reported in each tile. **c** MA plot of transcriptome profiling in FK866-treated vs. untreated CEM RES cells. For each gene, the average log_10_ signal against the log_2_ fold change is plotted. **d** CEM PA cells were treated with 5 and 100 nM FK866 for 48 h. Western blot showing expression of AMPK, mTOR, 4EBP1, and EIF2A in CEM PA cells. **e** No translation inhibition and drug-induced energetic stress occurs upon FK866 treatment in the resistance. Western blot analysis of CEM RES cells treated 100 nM FK866 for 48 h. Quantitative data are expressed as relative expression to MOCK (normalized by ACTIN). **f** CEM RES cells treated with 5 nM FK866 for 48 h. Expression of *CHOP* was determined. Quantitative data were derived from three independent experiments and expressed as the mean ± S.D. (***p < 0.001). **g** Barplot displaying genes belonging to the enriched metabolic pathways OXPHOS and glycolysis (KEGG annotation) and with significantly altered expression between CEM PA and CEM RES cells

To evaluate the effects of FK866 on gene expression, we profiled the transcriptome of parental vs. resistant CEM cells treated or not with the NAMPT inhibitor FK866 by microarrays. Using a double threshold on statistical significance (*p* value < 0.05) and log2 fold change (absolute value > 0.75), we identified 629 differentially expressed genes (DEGs, 285 upregulated and 344 downregulated) after FK866 treatment of parental cells (Fig. 3a, Additional file 3: Table S1). Functional annotation analyses of all DEGs highlighted the modulation of transcripts responsible for the epigenetic status of the cells. Specifically, we observed the enrichment of gene ontology (GO) categories associated with lysine acetylation; modulation of key epigenetic regulators, such as *ATF2*, *SIRT1*, and *SIRT3*; and transcription factors involved in energetic stress response and NAD(H) dependent enzymes. In addition, the enrichment of categories associated with cell response to DNA damage (*CHEK1*, *GADD45G*, *CHOP*) and modulation of apoptotic-converging signaling cascades suggested that FK866 caused toxic effects in parental cells (Fig. 3b, Additional file 3: Table S1). Exposure to FK866 of CEM RES did not induce significant changes in gene expression (about 150 DEGs, 66 upregulated and 96 downregulated), which is in line with the lack of effect of this agent on cell metabolism (Fig. 3c, Additional file 3: Table S1). Indeed, treatment of CEM RES did not activate the integrated stress response (ISR) as shown by the fact that 4EBP1 and EIF2A, two key proteins regulating translation initiation, did not change their phosphorylation status, suggesting that these pathways were not activated by FK866 treatment. Notably, AMPK, the AMP-regulated kinase sensing the cell energetic status and responding to stress, was constitutively phosphorylated in CEM RES, possibly suggesting that CEM RES rely on the activation of this kinase for overcoming the FK866 insult (Fig. 3d, e) [41]. *CHOP* expression also did not increase (Fig. 3f), thus showing that a key pro-apoptotic protein that became upregulated by FK866 in parental cells was not triggered in resistant cells by FK866 treatment. Direct comparison of untreated CEM RES vs. CEM PA highlighted major differences between the two samples. More than 5000 genes were differentially regulated (2354 upregulated, 2681 downregulated, Additional file 3: Table S1), a difference that clearly indicates a profound gene expression rewiring in resistant cells. According to a functional annotation of the transcripts undergoing major expression variations, strong differences were observed in metabolic pathways, such as glycolysis, oxidative phosphorylation (OXPHOS), NAD^+^ biosynthesis, and amino acid transport (Fig. 3g, Additional file 3: Table S1). No indication of differential expression of drug efflux pumps was observed, confirming that a multi-drug resistance mechanism is unlikely to mediate FK866 resistance in CEM. Taken together, gene expression analysis of CEM RES suggested that cancer cells adapted to FK866-induced NAD(H) depletion by adopting profound changes in gene expression and by modulating their metabolic pathways. Therefore, we focused on these metabolic changes and assessed whether compensating metabolic mechanisms would be responsible for conferring resistance to FK866 and could possibly highlight vulnerabilities of the resistant cells.

### CEM RES cells shift towards an aerobic glycolytic-based metabolism

**Fig. 4:**
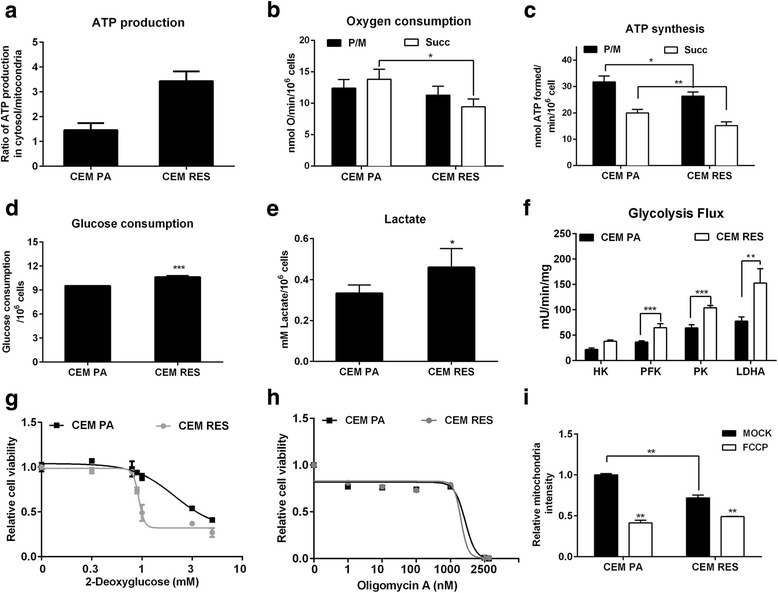
Enhanced glycolysis dependency of CEM RES cells. **a** Production of ATP from mitochondria and glycolysis were measured in CEM PA and CEM RES cells. Ratio of ATP production in the cytosol/mitochondria is shown. **b**–**f** Metabolic enhanced towards glycolysis in CEM RES cells; CEM PA and CEM RES subclones at 10, 40, and 100 nM of FK866 were determined the aerobic metabolism, oxygen consumption (**b**), and ATP synthesis (**c**). Cells were analyzed in the presence of 5 mM pyruvate + 2.5 mM malate (P/M) or 20 mM succinate (Succ) to stimulate the pathways composed by complexes I, III, and IV or complexes II, III, and IV, respectively. To measure the glycolytic flux, glucose consumption (**d**), lactate production (**e**), and the activity of hexokinase (HK), 6-phospho-fructokinase (PFK), pyruvate kinase (PK), and lactate dehydrogenase (LDH) (**f**) were analyzed. **g**–**h** CEM PA and CEM RES cells were treated with 2-deoxyglucose (**g**) and oligomycin A (**h**) for 48 h. Relative cell viability is shown. **i** Analysis of mitochondria content was conducted in CEM PA and CEM RES cells. Cells were exposed to mitotracker staining. FCCB was used as a negative control. Data was shown as mean ± S.D. of three independent experiments with technical triplicates, *p < 0.05, **p < 0.01, and ***p < 0.001

We evaluated the degree of activation of glycolysis and of OXPHOS in CEM RES by measuring the level of ATP production in the mitochondria and in the cytoplasm, as well as the activity of key OXPHOS and glycolytic enzymes. Cytosolic ATP production increased by about 15% in resistant cells (Fig. 4a), although the energy production efficiency of both pathways of the mitochondrial respiratory chain, the one including complexes I, III, and IV and the one comprising complexes II, III, and IV, was reduced in CEM RES. In particular, the oxygen consumption rate (OCR) appeared significantly low only after succinate addition (Fig. 4b) that stimulates the pathway led by complex II, while ATP synthesis by Fo-F1 ATP synthase was impaired both in the presence of pyruvate + malate (which induces the complex I pathway) and with succinate (Fig. 4c). Parental and resistant cells showed a coupled OXPHOS as the P/O rate was within standard values [36]. The decrease in OXPHOS activity was paralleled by an increase in glucose consumption and in lactate production (Fig. 4d, e). The activities of the glycolytic enzymes, hexokinase (HK), phosphofructokinase (PFK), pyruvate kinase (PK), and lactate dehydrogenase (LDH), were all significantly increased in CEM RES as compared to the parental cells (Fig. 4f). Therefore, these data suggested that activation of aerobic glycolysis compensates for the decrease in OXPHOS observed in the resistant cells. We then evaluated whether FK866-resistant cells were addicted to glycolysis. We found that CEM RES were more sensitive to the glycolysis inhibitor 2-deoxyglucose, than parental cells, while no difference was observed when cells were treated with the OXPHOS inhibitor oligomycin A (Fig. 4g, h). Notably, consistent with the observed decrease in OXPHOS, the fluorescent signal produced by tetramethylrhodamine (TMRE), a dye whose incorporation into the mitochondria is driven by the strength of the mitochondrial transmembrane potential, was largely decreased in resistant cells (Fig. 4i). Therefore, CEM RES cells appear to compensate for the decrease in OXPHOS activity by increasing the activity of the glycolytic pathway and by shifting towards a Warburg-like metabolism.

### CEM RES cells are sensitive to tryptophan deprivation

**Fig. 5:**
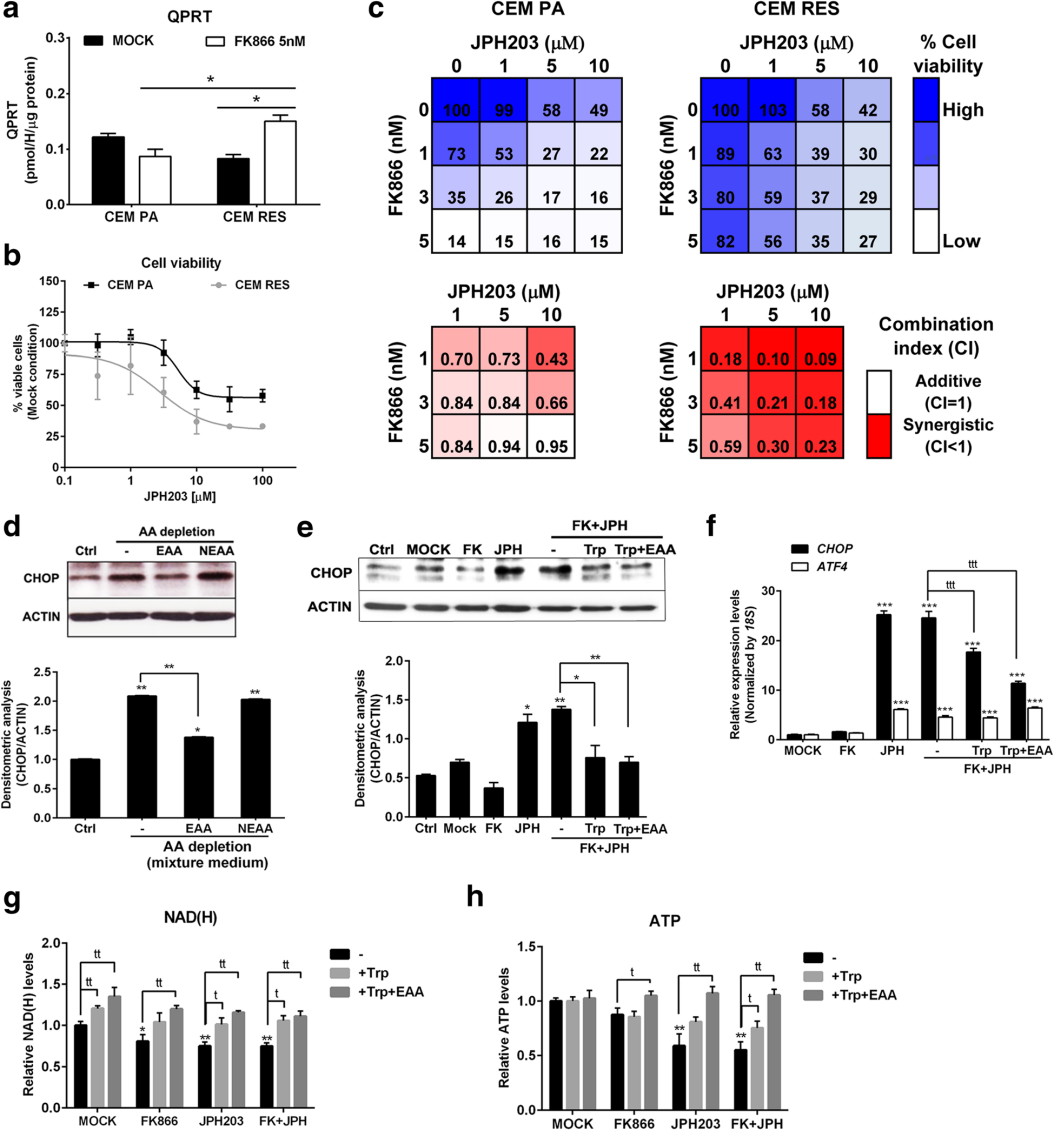
Tryptophan was served as an alternative source for NAD production in the resistant cells. **a** CEM PA and CEM RES cells were treated with 5 nM FK866 for 48 h. QPRT enzymatic activity was determined. **b** Percentage of cell viability indicates the response of CEM PA and CEM RES cells to JPH203 (LAT1 inhibitor) at 48 h of treatment. **c** Synergistic effect of FK866 and JPH203 in CEM cells. Strong synergistic effect as detected by low value of combination index (CI < 1) is more evident in CEM RES compared to CEM PA. Percentage of cell viability and CI are shown in respect to DMSO. **d** Activation of CHOP by amino acid deprivation. Western blot depicts the level of CHOP in CEM RES cells. CEM RES cells were treated with normal RPMI-1640 (10% FBS + 2 mM l-glutamine) (lane1), 20:80 mixture of normal RPMI-1640 (10% FBS + 2 mM l-glutamine) with Eagle’s balanced salt solution (10% FBS) (lanes 2–4) in the presence of essential amino acid (EAA) or non-essential amino acid (NEAA) for 24 h. **e** Tryptophan (Trp) rescues amino acid sensitized CEM RES cells to JPH203. This experiment was performed in normal RPMI-1640 (10% FBS + 2 mM l-glutamine) (lane 1) and mixture medium (lanes 2–6). CEM RES cells were treated with 5 nM FK866, 10 μM JPH203 (after O/N washout) or FK866 + JPH203. Supplementation of 0.25 mM tryptophan or 1× EAA was addressed to FK866 + JPH203 co-treatment. CHOP level was determined at 24 h after treatment. **f**–**h** CEM RES cells were treated with indicated conditions, and expression of *CHOP* and *ATF4* mRNA (**f**), NAD(H) level (**g**), and ATP level (**h**) were determined, respectively. Experiments in **a**–**c** were performed in the normal RPMI-1640 medium and in **d**–**h** were performed in the mixture medium. Data were plotted as mean ± S.D. from three independent experiments (*p < 0.05, ***p < 0.001 compared to MOCK) and (^tt^*p* < 0.01, ^ttt^*p* < 0.001 compared among treatments)

Thereafter, we measured the enzymatic activity of QPRT, NAPRT, and NRK (Fig. 1a), the enzyme converting nicotinamide riboside (NR) to NMN, in CEM cells to evaluate whether NAD^+^ biosynthesis  pathways alternative to the route controlled by NAMPT were exploited by CEM RES cells. As expected, no NAPRT activity was observed in both parental and resistant cells and no activity was measured after the FK866 treatment in both cell types. Interestingly, in resistant cells, NRK activity was not affected by FK866 treatment (not shown), whereas QPRT activity was modestly but significantly increased, suggesting that CEM RES cells may utilize amino acids as NAD^+^ precursors via the de novo QPRT-mediated NAD^+^ biosynthesis pathway to compensate for the diminished NAD^+^ synthesis via NAMPT (Fig. 5a). We also tested the activity of the enzymes nicotinamide mononucleotide adenylyltransferase (NMNAT), which converts NMN to NAD^+^ and nicotinic acid mononucleotide (NAMN) to nicotinic acid adenine dinucleotide (NAAD), and of glutamine-dependent NAD^+^ synthetase (NADS), which converts NAAD to NAD+. Both of these enzymes catalyze steps that are in common to both the de novo NAD^+^ biosynthesis pathway and the salvage routes from nicotinamide, nicotinic acid, and NR. No significant changes in the activity of these enzymes were observed (not shown). We subsequently tested whether CEM RES cells could use tryptophan (Trp) as an alternative NAD^+^ precursor to sustain NAD^+^ biosynthesis under stress conditions. To test this hypothesis, we assessed the activity of JPH203, an inhibitor of the L-type amino acid transporter 1 (LAT1) [30]. CEM RES cells were more sensitive to this drug than parental cells, showing a lower EC_50_ and a lower number of residual cells after treatment (Fig. 5b). In CEM PA, co-administration of JPH203 with FK866 showed additive effects at low doses of FK866 according to the calculation of the combination index (CI). In resistant cells, the combination of the two drugs had a synergistic effect in line with the higher efficacy of JPH203 in these cells than in parental cells (Fig. 5c). In amino acid deprivation experiments, CEM RES showed activation of the CHOP death pathway, indicating a fully active response of this stress-induced death pathway [41, 42]. Medium complementation with essential amino acids blocked CHOP activation while non-essential amino acids failed to do so (Fig. 5d). Therefore, CEM RES cells are sensitive to essential amino acid deprivation. Accordingly, co-treatment with JPH203 and FK866 activated the ATF4/CHOP death pathway, while this activation was prevented by tryptophan complementation, likely due to the competition of the amino acid with JPH203 for binding to LAT1 [43, 44] (Fig. 5e, f). Treatment with JPH203 and co-treatment with the two drugs caused a significant decrease in NAD(H) and ATP (Fig. 5g, h), showing the dependence of resistant cells on tryptophan metabolism for the production of these key metabolic molecules. Notably, the dependency on amino acid consumption appears to be caused by increased QPRT activity (Fig. 5a) and not by an increased expression of the LAT1 (Additional file 4: Figure S2C). Taken together, these data indicate that the development of FK866 resistance is associated with the addiction to at least one alternative mechanism of NAD^+^ production, namely, via tryptophan/QPRT pathway.

### CEM RES cells are sensitive to l-asparaginase treatment

**Fig. 6:**
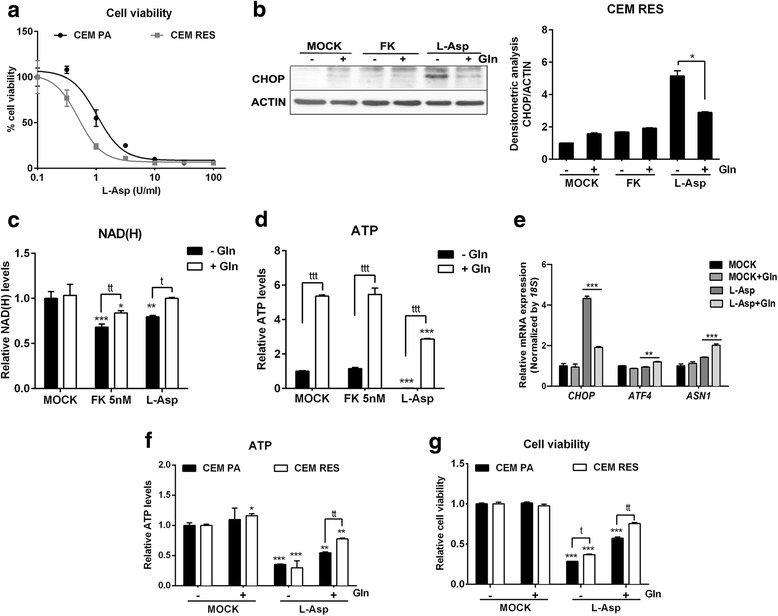
The resistant cells are sensitive to l-asparaginase. **a** CEM PA and CEM RES cells were treated with l-asparaginase (L-Asp) for 48 h. This experiment was performed in normal RPMI-1640 (10% FBS + 2 mM l-glutamine). Percentage of cell viability was determined. **b** CEM RES cells were treated with 5 nM FK866 and 3 U/ml L-Asp in the presence or absence of Gln supplementation. Twenty-four hours post-treatment, western blot analysis of CHOP expression was conducted. **c**, **d** CEM RES cells were treated with 5 nM FK866 and 3 U/ml L-Asp in the presence or absence of Gln supplementation. Twenty-four hours post-treatment, NAD(H) (**c**) and ATP level (**d**) were measured. **e** CEM RES cells were treated with 3 U/ml L-Asp in the presence and absence of Gln supplementation for 24 h. Expression of *CHOP*, *ATF4*, and *ASNS* were determined. Expression of *18S* was served as a housekeeping gene. **f**, **g** CEM PA and CEM RES cells were treated with 3 U/ml L-Asp in the presence and absence of Gln exposure for 24 h. ATP level (**f**) and cell viability (**g**) were measured. Experiments in **b**–**g** were performed in 20:80 mixture of normal RPMI-1640 (10% FBS + 2 mM l-glutamine) with Eagle’s balanced salt solution (10% FBS). Data were plotted as mean ± S.D. from three independent experiments with technical triplicates (*p < 0.05, **p < 0.01, ***p < 0.001 compared to MOCK) and (^tt^*p* < 0.01, ^ttt^*p* < 0.001 compared among treatments)

We also evaluated the importance of l-glutamine (Gln) in FK866-resistant cells. Gln is a non-essential amino acid that plays a fundamental role in cancer cell metabolism and promotes NAD^+^ production via NADS activity [45]. Specifically, we determined whether cancer cells with an acquired resistance to NAMPT inhibition were also more sensitive to Gln depletion than parental cells. To this end, we made use of l-asparaginase (L-Asp), an anticancer drug that degrades not only Asn but also Gln [46, 47]. Indeed, FK866-resistant CEM cells showed higher sensitivity to L-Asp than parental cells (Fig. 6a). The prevention of CHOP upregulation was obtained by Gln replenishment in FK866-resistant cells during treatment with L-Asp, confirming the importance of this amino acid for the survival of FK866-resistant cells (Fig. 6b). The same trend was observed when we monitored NAD(H) and ATP in CEM RES treated with the same agents (Fig. 6c, d). Interestingly, Gln also increased the expression level of the enzyme *ASNS* (Fig. 6e), which represents a possible protective mechanism [48]. Finally, Gln rescued L-Asp toxic effect and prevented the drop in ATP levels more evidently in CEM RES than in CEM PA (Fig. 6f, g). Overall, these data indicate that FK866-resistant CEM RES cells are relying on the presence of Gln in the medium to counteract FK866-induced reduction of NAD(H).

### FK866-resistant MDA MB231 have lower endogenous NAD(H) levels than parental cells but do not shift towards aerobic glycolysis

**Fig. 7:**
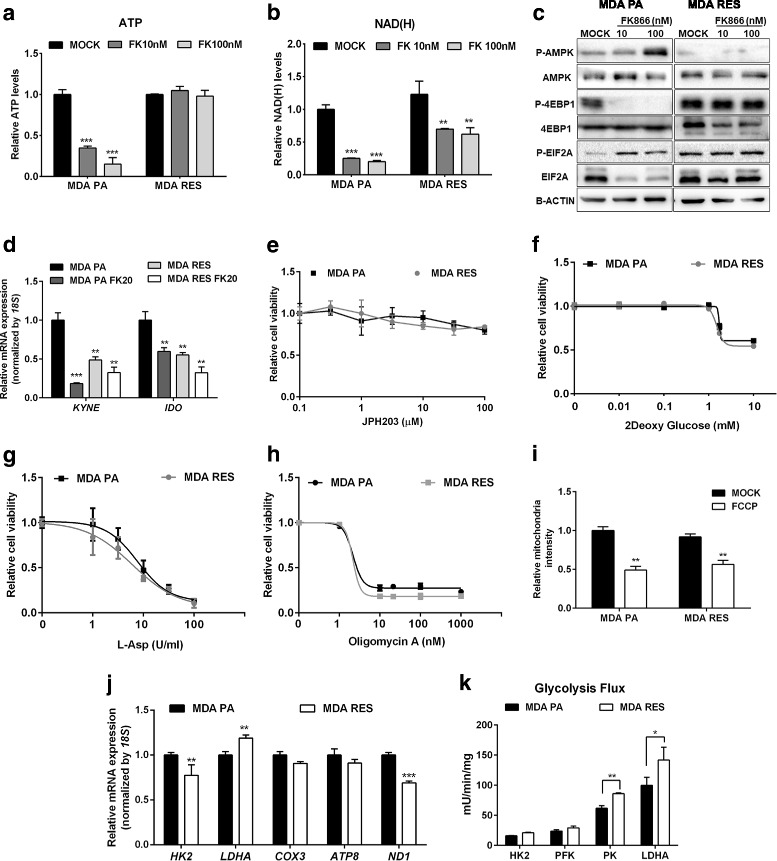
Characterization of MDA MB231 and FK866-resistant MDA MB231 cells. **a**, **b** MDA MB231 parental (MDA PA) and resistant cells (MDA RES) cells were treated with 10 and 100 nM FK866 for 48 h. ATP (**a**) and NAD(H) (**b**) levels were measured. **c** Western blot showing no alter in translation inhibition in MDA RES along with FK866 treatment in respect to MDA PA. Protein expression from total lysates were determined by detecting with indicated antibodies. B-ACTIN was used as a loading control. **d** MDA PA and MDA RES cells were treated with 20 nM FK866 for 48 h. Expression of *KYNE* and *IDO*, genes involved in the de novo pathway for NAD^+^ synthesis, were evaluated. **e**–**h** Relative cell viability of CEM PA and CEM RES cells treated with JPH203 (**e**), 2-deoxyglucose (**f**), L-Asp (**g**), and oligomycin A (**h**) for 48 h was displayed. **i** Analysis of mitochondria content. MDA PA and MDA RES cells were exposed to mitotracker staining. FCCB was used as a negative control. **j** Expression of genes involved in glycolysis and OXPHOS pathway were determined in MDA PA and MDA RES cells. Expression of *18S* was served as a housekeeping gene. **k** Enhanced LDHA activity in the resistance. Enzymatic activity of glycolysis pathway was measured in MDA PA and MDA RES cells. Data were plotted as mean ± S.D. from three biological experiments with technical triplicates (*p < 0.05, **p < 0.01, ***p < 0.001)

Similar to CEM RES, FK866-resistant MDA MB231 (MDA RES) were completely insensitive to CHS-828, again showing the independence of their resistance mechanism from the type of NAMPT inhibitor (Additional file 2: Figure S1E). In addition, no mutation was detected in the coding region of the *NAMPT* gene (not shown). ATP levels did not change during FK866 treatment in MDA RES (Fig. 7a), and NAD(H) level decreased to about 50% of the initial value likely due to NAMPT downregulation but did not drop as dramatically as it did in parental cells (Fig. 7b). The absence of toxicity of FK866 treatment in MDA RES cells was also shown by the continuous activation of the translation machinery that was not affected by the stress response inhibition mediated by EIF2A, AMPK, or 4EBP1 (Fig. 7c). However, as compared to parental cells (MDA PA) and to CEM RES cells, we observed several differences in terms of expression of NAD^+^-producing enzymes in MDA RES. In MDA MB231 parental cells, FK866 treatment strongly decreased the expression level of the enzymes involved in the de novo pathway for NAD^+^ biosynthesis, such as *IDO* and *KYNE* (Fig. 7d). The same enzymes were permanently downregulated in resistant cells and were further downregulated during FK866 treatment (Fig. 7d). Overexpression of NAMPT in resistant cells allowed the recovery of intracellular NAD(H) levels but did not re-sensitize cells to FK866 (Additional file 4: Figure S2D, E), suggesting that MDA RES cells developed a stable resistance mechanism that is independent from NAMPT-based NAD^+^ biosynthesis. This observation also suggested that MDA RES cells, similar to CEM RES, may use alternative biochemical pathways for NAD^+^ production. However, in this case, the de novo pathway was unlikely to be utilized since the enzymes involved in this metabolic route were found to be downregulated compared to the parental cells. In line with these findings, MDA RES cells did not show an increased sensitivity to amino acid deprivation, including tryptophan, as shown by their response to JPH203 (Fig. 7e). In addition, MDA RES also failed to show an increased sensitivity to 2-deoxyglucose, to L-Asp, and to oligomycin A (Fig. 7f–h), indicating the absence of a shift towards a Warburg-like metabolism or dependencies on specific amino acids. No difference in mitochondrial transmembrane potential (TMRE signal) was detected between parental MDA and MDA RES (Fig. 7i). As compared to CEM RES, in MDA RES, the expression level of OXPHOS genes was either unchanged, as in the case of the mitochondrial genes *COX3* and *ATP8*, or affected to a much lesser extent, as in the case of *ND1* (Fig. 7j, Fig. 3g, Additional file 4: Figure S2F). *HK2* gene expression was marginally downregulated in MDA RES (Fig. 7j), while its enzymatic activity did not change (Fig. 7k). On the other hand, both PK and LDHA were found to have increased enzymatic activity in MDA RES as compared to the parental cells. Both MDA PA and MDA RES were not sensitive to NAPRT inhibition with 2-hydroxynicotinic acid (2HNA), and *NAPRT* silencing did not sensitize resistant cells to FK866 (Additional file 4: Figure S2G, H, I, J). Therefore, MDA RES cells exhibit reduced levels of NAD^+^-producing enzymes from the de novo NAD^+^ production route. Different from CEM leukemia cells, they do not rely on QPRT as an alternative NAD^+^-producing enzyme and NAPRT-mediated NAD^+^ biosynthesis also seems not be involved in their resistance phenotype.

### LDHA is the key enzyme regulating the metabolic resistance to NAMPT inhibition

**Fig. 8:**
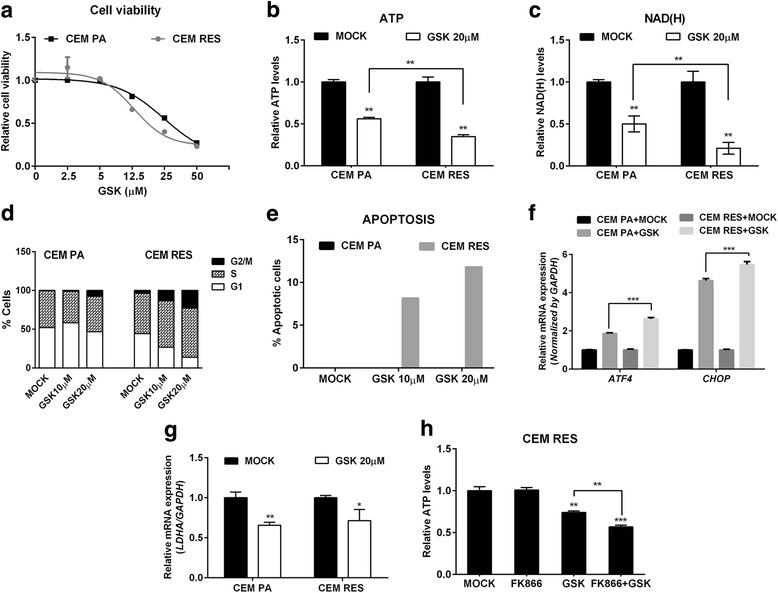
LDHA regulates metabolic resistance in CEM cells. **a** CEM PA and CEM RES cells were treated with LDH inhibitor (GSK) for 48 h. Relative cell viability was analyzed using MTT assay in respect to Mock. **b**, **c** CEM PA and CEM RES cells were treated with 20 μM GSK for 48 h; ATP (**b**) and NAD(H) (**c**) levels were determined. **d**, **e** CEM PA and CEM RES cells were treated with 10 and 20 μM GSK for 48 h. Cell cycle analysis and percentage of apoptotic cell were determined, respectively. **f**, **g** CEM PA and CEM RES cells were treated with 20 μM GSK for 48 h; quantitative RT-PCR of *ATF4*/*CHOP* (**f**) and *LDHA* (**g**) was determined, respectively. Expression of *GAPDH* was used as a housekeeping gene. **h** Synergistic effect of FK866 and GSK was detected by a strong reduction of ATP level in CEM RES cells. ATP level was measured in CEM RES cells treated with 5 nM FK866, 20 μM GSK, and co-treatment between FK866 and GSK for 48 h. All data were represent as mean ± S.D. from three independent experiments (*p < 0.05, **p < 0.01, ***p < 0.001)

**Fig. 9:**
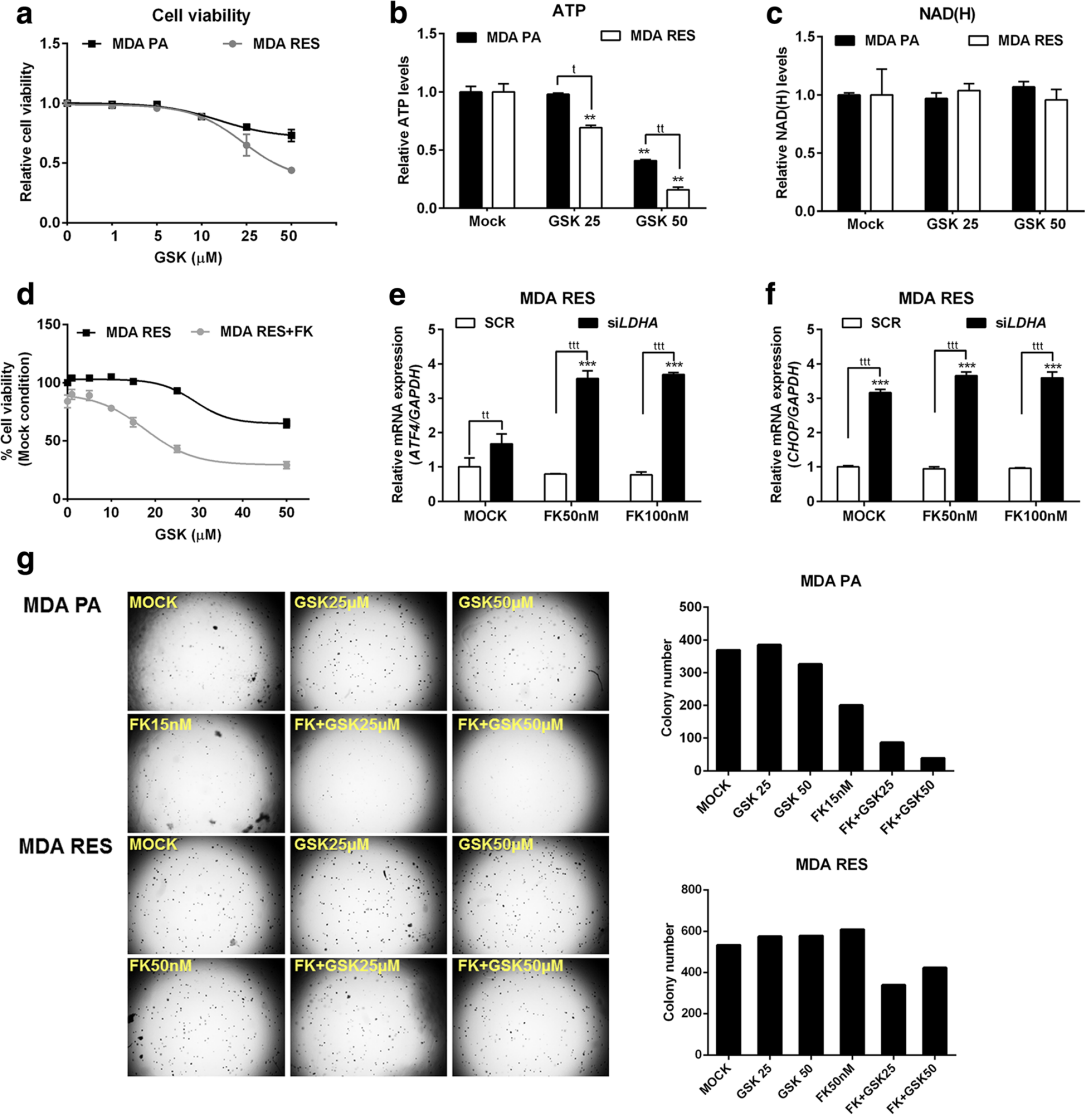
Pharmacological and genetic inhibition of LDHA in MDA MB231 cells. **a** MDA PA and MDA RES cells were treated with GSK for 48 h. Relative cell viability was analyzed. **b**, **c** MDA PA and MDA RES cells were treated with 25 and 50 μM GSK for 48 h; ATP (**b**) and NAD(H) (**c**) levels were determined, respectively. **d** Synergistic effect of FK866 and GSK inhibits cell growth in MDA RES cells. MDA RES cells were treated with various concentrations of GSK and co-treatment with 20 nM FK866 for 48 h. Percentage of cell viability was determined. **e, f** Downregulation of *LDHA* by siRNA was performed in MDA RES cells. Twenty-four hours after silencing, MDA RES cells were treated with GSK for 48 h. Expression levels of *ATF4* (**e**) and *CHOP* (**f**) were evaluated. Expression of *GAPDH* was used as a housekeeping gene. All data were represent as mean ± S.D. from three independent experiments (*p < 0.05, **p < 0.01, ***p < 0.001). **g** MDA PA and MDA RES cells were performed anchorage-independent growth (colony formation assay) in soft agar along with indicated concentrations of FK866 and GSK treatment for 21 days. A representative colony formation experiment was illustrated and colony number was counted by operetta

LDHA expression and enzymatic activity were both increased in MDA RES (Fig. 7j, k), as well as in CEM RES (Fig. 4f), suggesting that LDHA activity may be not only a common mechanism of acquired resistance to NAMPT inhibition but also a new possible vulnerability of FK866-resistant cancer cells. To evaluate the importance of LDHA in the resistance of both cell models, we treated our cell lines with the LDH inhibitor GSK2837808A (GSK). When using GSK as a single agent, we observed a marginal increase in sensitivity in CEM RES compared to parental cells (Fig. 8a) and a decrease in ATP and NAD(H) levels (Fig. 8b, c). In cell cycle experiments, we found that GSK induced a strong accumulation in the S and in the G2/M phases of the cell cycle in CEM RES cells (Fig. 8d, e) [49]. At the molecular level, GSK activated the ATF4/CHOP-dependent pro-apoptotic pathway and decreased lactate production (Fig. 8f and Additional file 5: Figure S3A). GSK also decreased *LDHA* expression (Fig. 8g), in addition to blunting its enzymatic activity, and effectively re-sensitized CEM RES cells to FK866 in co-treatment experiments as shown by an additive ATP decrease (Fig. 8h). MDA RES cells showed a similar but not identical response to GSK treatment. Specifically, MDA RES were more sensitive than the parental population to GSK treatment at high doses (Fig. 9a, b). GSK effectively reduced the acidity of the medium both in MDA PA and in MDA RES (Additional file 5: Figure S3B). By itself, GSK did not decrease NAD(H) levels and it did not activate the ATF4/CHOP apoptotic pathway in MDA RES (Fig. 9c, Additional file 5: Figure S3C, D). However, it effectively re-sensitized MDA RES cells to FK866 in co-treatment experiments (Fig. 9d). To prove the relevance of LDHA in determining resistance to FK866, we silenced it by RNA interference with siRNAs (Additional file 5: Figure S3E, F) and exposed the cells to FK866 treatment. Indeed, LDHA silencing phenocopied the effect of GSK treatment by reducing ATP but not NAD(H) levels, decreasing lactate production and medium acidity (Additional file 5: Figure S3G, H, I)*.* In response to *LDHA* silencing, FK866 also activated the pro-apoptotic ATF/CHOP pathway (Fig. 9e, f), which is in line with the synergistic effect observed during co-treatment with GSK. In MDA PA, GSK potentiated FK866’s ability to reduce the clonogenic capacity of these cells. MDA RES cells were sensitive only to the co-treatment with GSK and FK866 but were fully resistant to the single treatments (Fig. 9g). Therefore, overall, these data indicate that LDHA plays a pivotal role in the metabolic rewiring that underlies cancer cells’ acquired resistance to NAMPT inhibition in our models.

## Discussion

**Fig. 10:**
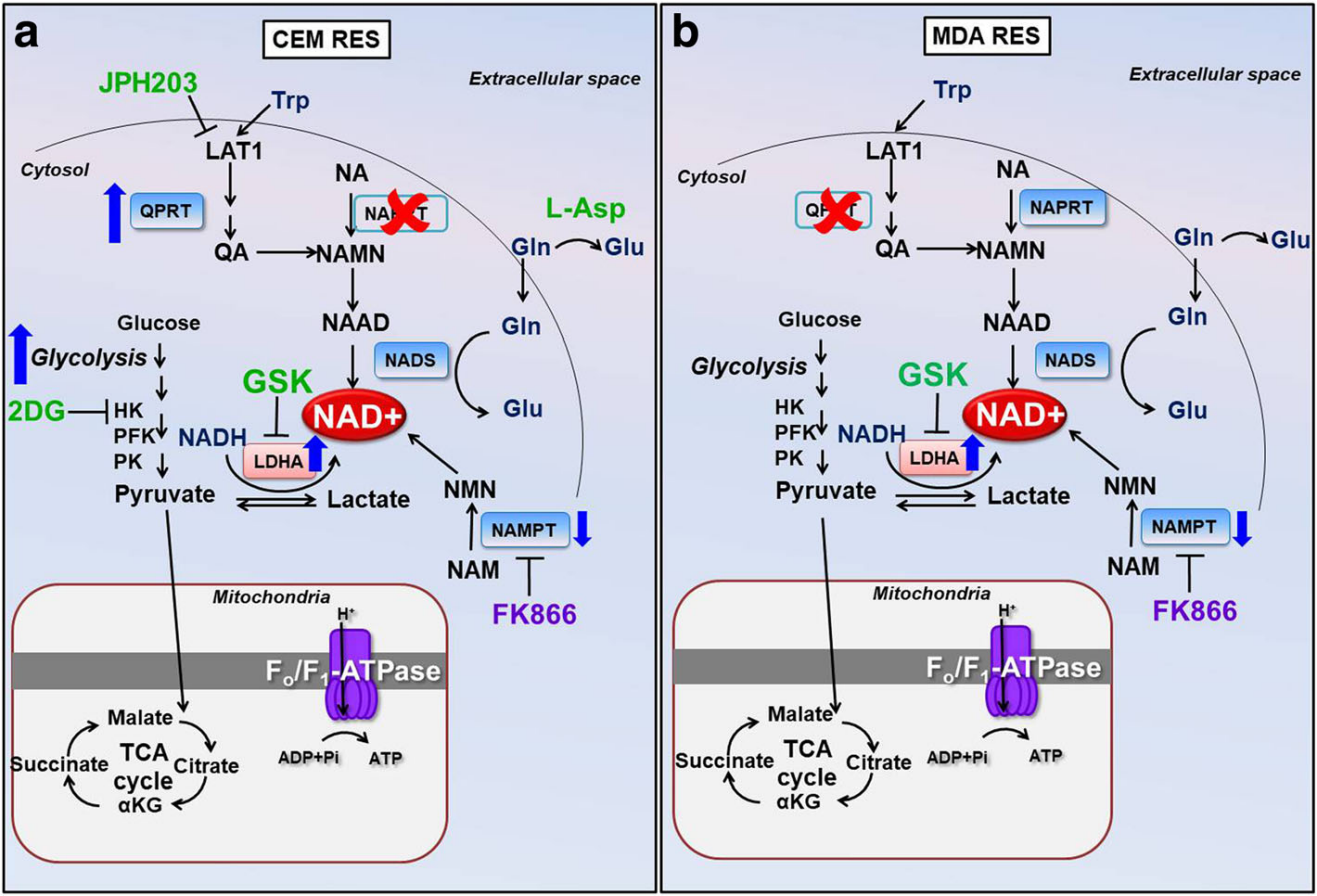
Schematic representation of different metabolic responses in acquiring FK866 resistance in CEM RES (**a**) and MDA RES (**b**) cells. CEM RES cells are NAPRT-negative and QPRT-positive cells and appear to increase the de novo pathway for NAD production. The metabolic resistance of CEM is more dependent on glycolysis and LDHA activity whereas MDA RES shows very low level of QPRT and primarily rely on LDHA activity for its resistance to FK866. The small molecules having a pro-death pharmacological effect are indicated in green. Abbreviations: NAD, nicotinamide adenine dinucleotide; NA, nicotic acid; NAMN, nicotinic acid mononucleotide; NAAD, nicotinic acid adenine dinucleotide, NAPRT, nicotinic acid phosphoribosyltransferase; NMNAT, nicotinamide mononucleotide adenylyltransferase; NADS, NAD synthase; NAM, nicotinamide; NMN, nicotinamide mononucleotide; NAMPT, nicotinamide phosphoribosyltransferase; NR, nicotinamide riboside; NRK, nicotinamide riboside kinase; QPRT, quinolinate phosphoribosyltransferase; HK, hexokinase; PFK, phosphofructokinase; PK, pyruvate kinase; LDH, lactate dehydrogenase; Gln, glutamine; Glu, glutamic acid; GSK, LDHA inhibitor; 2DG, 2-deoxyglucose; JPH, JPH203; L-Asp, l-asparaginase; GSK, GSK2837808A; LAT1, L-type amino acid transporter 1

In this study, we applied a selection strategy to obtain T-ALL and triple-negative breast cancer cells that are resistant to the NAMPT inhibitor FK866 and essentially observed that the metabolic plasticity of cancer cells allows them to escape the toxic insult of this drug after protracted exposure. According to their different initial genetic background, the two cell lines that we utilized for our study, CCRF-CEM and MDA-MB231, exploit different mechanisms to synthesize NAD^+^: the T-ALL cell line, CCRF-CEM, was found not to express NAPRT but expressed QPRT, and their resistance phenotype reflected the ability to exploit amino acid catabolism, on the one hand, and an increase in their glycolytic flow and LDHA activity, on the other. The triple-negative breast cancer cell line, MDA MB231, was found to express NAPRT, to have low levels of QPRT, and to primarily rely on LDHA activity for its resistance to FK866. Importantly, in both cell models, cancer cell death appeared to occur via reactivation of the EIF2A/ATF4/CHOP pathway (Fig. 10).

Chronic exposure to an anticancer agent frequently leads to the development of resistance [1, 2], and this notion also applies to metabolic drugs [50]. In the case of NAMPT inhibitors, most of the mechanisms of resistance described so far consist in mutations in the enzyme itself with consequent loss of protein/drug interaction [28]. In our models, we found that the acquisition of resistance to the NAMPT inhibitor FK866 was not caused by mutations in *NAMPT* but rather reflected by a metabolic rewiring. Our selection protocol was based on the quick adaptation of resistant cells to small drug increases [40], reducing at minimum the possibility to introduce gross genetic rearrangements and thus favoring the escape of pre-existing resistant cells [8]. Notably, we found that the acquisition of resistance to FK866 exposed new, specific cell vulnerabilities. At the molecular level, FK866-resistant cancer cells were found to effectively prevent the activation of the EIF2A/ATF4/CHOP pathway that promotes cell death [41, 51, 18]. Microarray gene expression analyses supported by biochemical evaluations of metabolites (Fig. 4f) suggested that FK866-resistant T-ALL cells pushed glycolysis at the expenses of OXPHOS (Fig. 3g), rendering resistant cells more susceptible to anti-glycolytic drugs (Fig. 4g). In addition, the need to produce NAD^+^ via an alternative biochemical path (in a context in which NAPRT is not expressed) (Fig. 1a, c) translated into a dependency on amino acids such as tryptophan and glutamine. Notably, a dependency on QPRT activity was recently observed in human fibrosarcoma cells with acquired resistant to NAMPT inhibition that still presented a point mutation in the NAMPT enzyme [29]. We specifically investigated tryptophan and glutamine for their role in acquired resistance to NAMPT inhibitors. Tryptophan is the NAD^+^ precursor utilized in the “de novo” NAD^+^ biosynthesis pathway. We observed that T-ALL cells that are resistant to FK866 rely on tryptophan for NAD^+^ production, which indicated a possible metabolic vulnerability of these cells. JPH203, an inhibitor of amino acid transport, was shown to possess anticancer activity both in vitro and in vivo against HT-29 tumors transplanted in nude mice [44, 52] and to cause apoptosis in YD-38 human oral cancer cells [53]. In our hands, this compound induced CHOP activation in FK866-resistant CCRF-CEM cells. Interestingly, the same was found to hold true for Gln [54], another amino acid involved in the production of NAD^+^, not as an NAD^+^ precursor but rather as a nitrogen donor in the reaction catalyzed by the enzyme NAD synthetase (*NADS*). Gln degradation by L-Asp also activated CHOP and potentiated FK866 activity in FK866-resistant CCRF-CEM. Thus, essential amino acid utilization emerges as an alternative source for NAMPT-independent NAD^+^ production in FK866-resistant T-ALL cells. The triple-negative breast cancer model represented by MDA MB231 cells expresses NAPRT, an enzyme that was previously associated to resistance to FK866 [55]. In this model, we observed different mechanisms of acquired resistance to FK866. Specifically, as compared to parental cells, MDA RES cells failed to show changes in their balance between glycolysis and OXPHOS or increased dependency on amino acids (Fig. 7e–h). NAPRT also did not seem to play a role in MDA RES metabolism in baseline conditions. Importantly, both FK866-resistant CCRF-CEM and MDA MB231 showed increase LDHA activity, and this enzyme emerged as a pivotal protein for mediating FK866 resistance. High *LDHA* levels have been linked to poor prognosis in many cancers, including liver and lung cancer [56]. Downregulation of *LDHA* in cancer cells by siRNA or shRNA stimulates mitochondrial respiration and reduces cellular proliferative and tumorigenic potential in cancer cells in vitro and in tumor xenografts [57–59]. In highly glycolytic tumors, such as lymphoma, the combination of an LDHA inhibitor and a NAMPT inhibitor resulted in a synergistic anticancer effect [60]. Moreover, breast cancer cells with acquired resistance to taxol were re-sensitized to this chemotherapeutic by inhibiting LDHA [61]*.* Consistent with these studies, we found that the combination of FK866 with GSK was highly effective against FK866-resistant CCRF-CEM. LDHA silencing in MDA RES cells increased their sensitivity to FK866 as shown by CHOP/ATF4 activation. Thus, targeting LDHA together with NAMPT may offer novel opportunities for selective killing of cancer cells, particularly in cancer types that are addicted to aerobic glycolysis for survival.

## Conclusions

In conclusion, our data show that FK866 treatment can lead to the development of different mechanisms of acquired resistance. Such mechanisms depend on the original metabolic profile of the tumor, including the pattern of expression of NAD^+^-producing enzymes, such as NAPRT or QPRT, since the latter can come into play for compensating for chronic NAMPT inhibition, ensuring that sufficient NAD^+^ is produced for a cancer cell to survive. Closely monitoring the metabolic adaptations of a tumor to NAMPT inhibition could be useful to inform therapeutic decisions. In this context, LDHA inhibitors should be considered for further evaluation in clinical trials as drugs capable of reverting resistance to NAMPT inhibitors.

## Additional files

Additional file 1: **Table S2.** List of RT-PCR primer sequences. (XLS 34 kb)

Additional file 2: **Figure S1.** A Dose-dependent resistant to FK866; CEM PA and CEM RES cells at 100 nM of FK866 were treated with various concentrations of FK866 for 48 h. Percentage of cell viability was conducted by MTT assay and analyzed in respect to DMSO treatment. B CEM PA and CEM RES 100 nM cells were treated with 5 or 100 nM of FK866 for 72 h. Percentage of viable and dead cells was evaluated by DAPI staining and analyzed in respect to DMSO. C CEM RES cells were insensitive to long-term treatment of FK866; cells were grown in the present of 500 nM FK866 for 5 days. Percentage of cell viability was measured by 0.2% trypan blue staining. D, E Resistant cells were insensitive to another NAMPT inhibitor (CHS-828). CEM and MDA cells were treated with CHS-828 for 48 h. Percentage of cell viability was analyzed. F-H CEM PA and CEM RES cells were treated with 5 nM FK866 for 48 h. Amount of ATP (represent as energy charge), NAD+, and NMN levels were evaluated through HPLC analysis and referred to the protein content. I Cell cycle analysis was conducted in CEM PA and CEM RES cells by FACS analysis. J No involvement of multi-drug resistance mechanism in the resistant CEM model; CEM PA and CEM RES cells were treated with 2.5 nM FK866, 5 μM cyclosporin A (CsA), 5 μM verapamil, and combination of FK866 with CsA or verapamil for 48 h. Relative cell viability was accessed by MTT assay. (PDF 1757 kb)

Additional file 3: **Table S1.** List of gene expression from genome wide analysis in CCRF-CEM cells. (XLSX 44303 kb)

Additional file 4: **Figure S2.** A CEM RES cells were maintained in the absence of FK866 (a washout condition) for 2 months as referred to CEM RES WO; then, they were re-exposed to 100 nM FK866 for 96 h. Relative ATP level was measured. B CEM PA, CEM RES with FK866 (CEM RES), and CEM RES-washed out FK866 (CEM WO FK) cells were re-exposed to 100 nM FK866 for 48 h. Relative ATP levels were measured at 48 h after the treatment. C Expression of L-type amino acid transporter 1 (LAT1) in parental and resistant cells. Expression of LAT1 in CCRF-CEM and MDA MB231 cells was determined by CD98 staining and the analysis was performed by FACS. D–E MDA PA and MDA RES cells were transiently transfected with two different NAMPT overexpressed plasmids (NAMPT1 and NAMPT2) or empty vector (PBP). Twenty-four hours after transfection, cells were treated with 20 and 40 nM FK866 for 48 h. NAD(H) levels (D) and cell viability (E) were determined. F Expression of gene involved glycolysis and OXPHOS pathways in CEM PA and CEM RES cells. G MDA PA and MDA RES cells were treated with a NAPRT inhibitor (2-HNA) for 48 h. Relative cell viability compared to untreated control was evaluated by  OZBlue Cell Viability kit. H Stable downregulation of *NAPRT* cell lines were obtained from transduction of lentiviral vector contained sh*NAPRT*. Western blot depicts decrease in NAPRT levels of sh*NAPRT* MDA cells. GAPDH was used as a loading control. I–J Stable sh*NAPRT* MDA PA and MDA RES cell lines were treated with 20 nM FK866 in MDA PA and 100 nM FK866 in MDA RES for 48 h. Relative ATP (I) and NAD(H) levels (J) compared to MOCK were determined (*p < 0.05, **p < 0.01 compared to MOCK). (PDF 2199 kb)

Additional file 5: **Figure S3.** A MDA PA and MDA RES cells were treated with 25 and 50 μM GSK for 48 h. Lactate production was determined. B Reduction of acidity was observed in GSK treatments. MDA PA and MDA RES cells were treated with various concentrations of GSK (0.1–50 μM) for 48 h. A decrease of acidity was visualized by changing of medium colors from red to yellow (basic to acidic). C, D Expression of *ATF* and *CHOP* mRNA along with 25 μM GSK treatment for 48 h in MDA. E Western blot showing downregulation of LDHA level in LDHA silencing MDA cells conducted by siRNA. ACTIN was used as a loading control. F FK866 decreased *LDHA* mRNA levels. MDA RES cells were transiently transfected with siLDHA. Twenty-four hours after silencing, cells were treated with 50 and 100 nM FK866 for 48 h. Expression of *LDHA* was detected. G–I MDA PA and MDA RES cells were transiently transfected with siLDHA. Forty-eight hours post transfection, ATP (G), NAD(H) (H), and lactate production (I) were measured (*p < 0.05, **p < 0.01, ***p < 0.001). (PDF 2727 kb)

## Abbreviations

2HNA: 2-Hydroxynicotinic acid
CEM PA: Parental CCRF-CEM cells
CEM RES: FK866-resistant CCRF-CEM cells
CEM: CCRF-CEM cells
DEGs: Differentially expressed genes
GSK: LDH inhibitor GSK2837808A
HK: Hexokinase
L-Asp: l-Asparaginase
LAT1: L-type amino acid transporter 1
LDHA: Lactate dehydrogenase A
LVP: Lentiviral vector particle
MDA RES: FK866-resistant MDA MB231 cells
MDA PA: Parental MDA MB231 cells
MDR: Multi-drug resistance
NA: Nicotinic acid
NAMN: Nicotinic acid mononucleotide
NAMPT: Nicotinamide phosphoribosyltransferase
NAPRT: Nicotinic acid phosphoribosyltransferase
OCR: Oxygen consumption rate
OXPHOS: Oxidative phosphorylation
PFK: Phosphofructokinase
PK: Pyruvate kinase
QPRT: Quinolinate phosphoribosyltransferase
TMRE: Tetramethylrhodamine
TRAIL: Tumor necrosis factor-related apoptosis-inducing ligand
Trp: Tryptophan
