# Supplementary figures and images for: Cancer cell metabolic plasticity allows resistance to NAMPT inhibition but invariably induces dependence on LDHA
Source: Cancer Metab. 2018 Mar 8;6:1. doi: 10.1186/s40170-018-0174-7 (PMC5844108; doi:10.1186/s40170-018-0174-7)

# Additional Figure 1

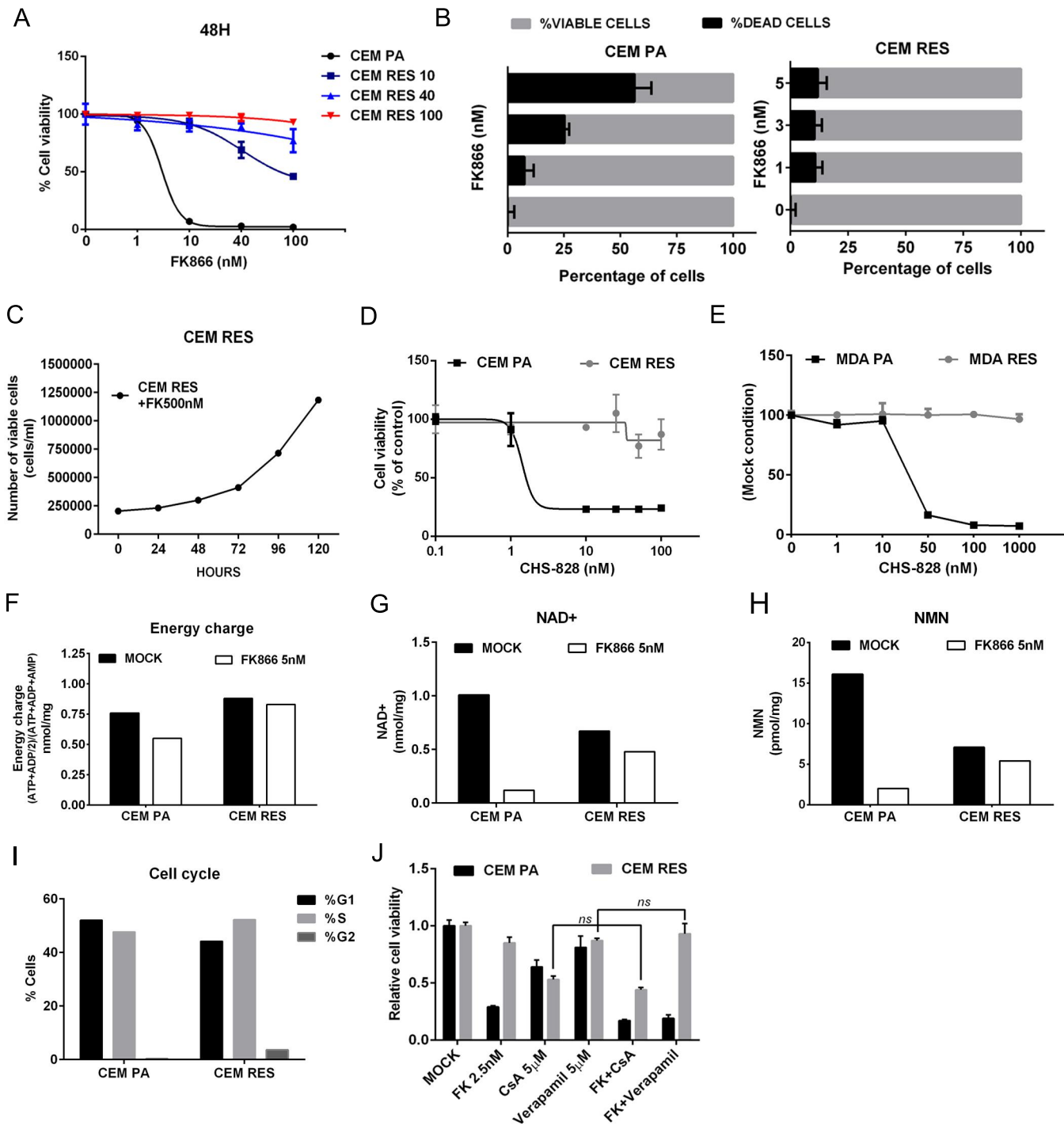

Supplement: Supplementary file 2 — Figure S1. A Dose-dependent resistant to FK866; CEM PA and CEM RES cells at 100 nM of FK866 were treated with various concentrations of FK866 for 48 h. Percentage of cell viability was conducted by MTT assay and analyzed in respect to DMSO treatment. B CEM PA and CEM RES 100 nM cells were treated with 5 or 100 nM of FK866 for 72 h. Percentage of viable and dead cells was evaluated by DAPI staining and analyzed in respect to DMSO. C CEM RES cells were insensitive to long-term treatment of FK866; cells were grown in the present of 500 nM FK866 for 5 days. Percentage of cell viability was measured by 0.2% trypan blue staining. D, E Resistant cells were insensitive to another NAMPT inhibitor (CHS-828). CEM and MDA cells were treated with CHS-828 for 48 h. Percentage of cell viability was analyzed. F-H CEM PA and CEM RES cells were treated with 5 nM FK866 for 48 h. Amount of ATP (represent as energy charge), NAD+, and NMN levels were evaluated through HPLC analysis and referred to the protein content. I Cell cycle analysis was conducted in CEM PA and CEM RES cells by FACS analysis. J No involvement of multi-drug resistance mechanism in the resistant CEM model; CEM PA and CEM RES cells were treated with 2.5 nM FK866, 5 μM cyclosporin A (CsA), 5 μM verapamil, and combination of FK866 with CsA or verapamil for 48 h. Relative cell viability was accessed by MTT assay. (PDF 1757 kb) [file 40170_2018_174_MOESM2_ESM.pdf]

# Additional Figure 2

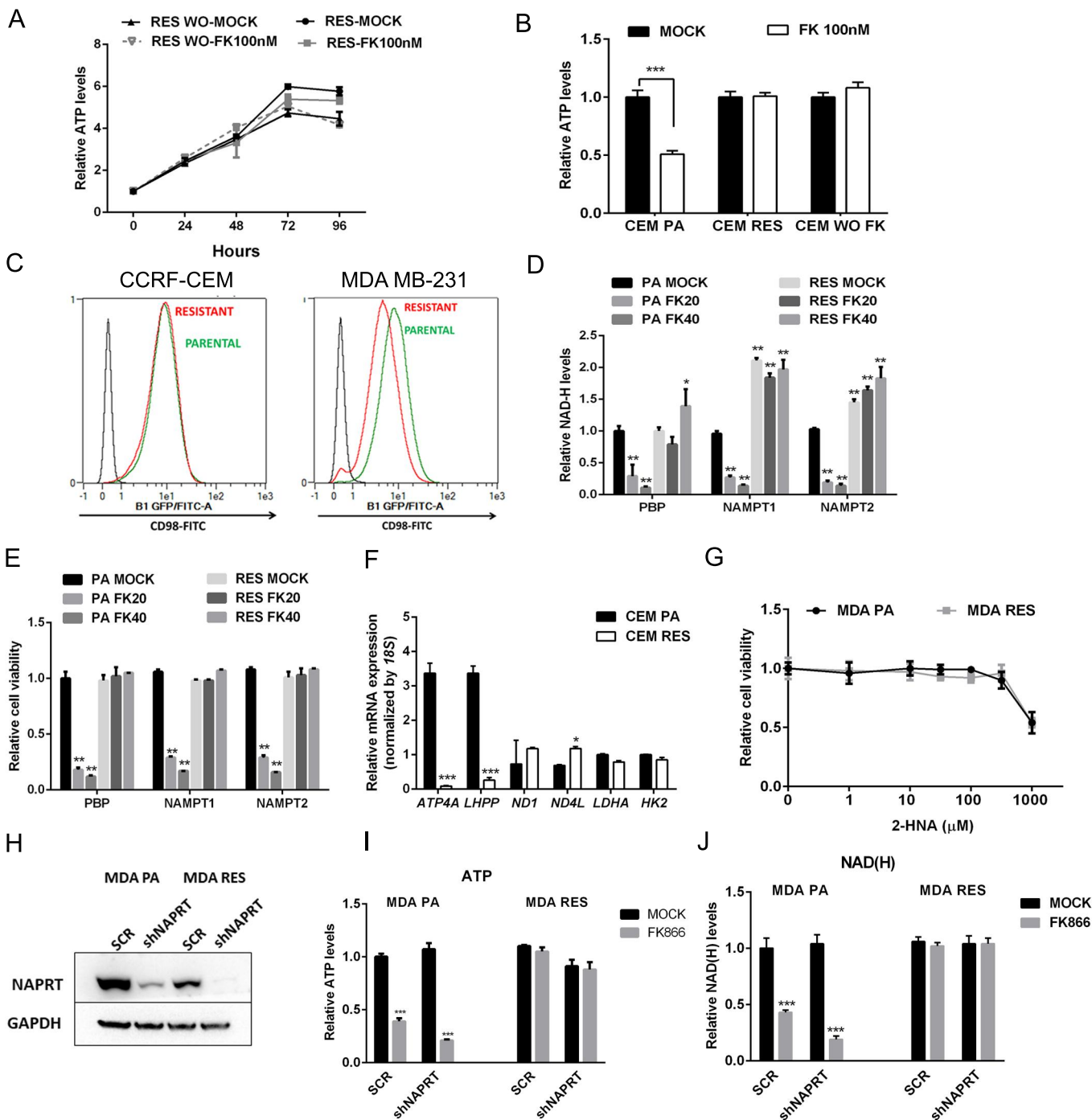

Supplement: Supplementary file 4 — Figure S2. A CEM RES cells were maintained in the absence of FK866 (a washout condition) for 2 months as referred to CEM RES WO; then, they were re-exposed to 100 nM FK866 for 96 h. Relative ATP level was measured. B CEM PA, CEM RES with FK866 (CEM RES), and CEM RES-washed out FK866 (CEM WO FK) cells were re-exposed to 100 nM FK866 for 48 h. Relative ATP levels were measured at 48 h after the treatment. C Expression of L-type amino acid transporter 1 (LAT1) in parental and resistant cells. Expression of LAT1 in CCRF-CEM and MDA MB231 cells was determined by CD98 staining and the analysis was performed by FACS. D–E MDA PA and MDA RES cells were transiently transfected with two different NAMPT overexpressed plasmids (NAMPT1 and NAMPT2) or empty vector (PBP). Twenty-four hours after transfection, cells were treated with 20 and 40 nM FK866 for 48 h. NAD(H) levels (D) and cell viability (E) were determined. F Expression of gene involved glycolysis and OXPHOS pathways in CEM PA and CEM RES cells. G MDA PA and MDA RES cells were treated with a NAPRT inhibitor (2-HNA) for 48 h. Relative cell viability compared to untreated control was evaluated by OZBlue Cell Viability kit. H Stable downregulation of NAPRT cell lines were obtained from transduction of lentiviral vector contained shNAPRT. Western blot depicts decrease in NAPRT levels of shNAPRT MDA cells. GAPDH was used as a loading control. I–J Stable shNAPRT MDA PA and MDA RES cell lines were treated with 20 nM FK866 in MDA PA and 100 nM FK866 in MDA RES for 48 h. Relative ATP (I) and NAD(H) levels (J) compared to MOCK were determined (*p < 0.05, **p < 0.01 compared to MOCK). (PDF 2199 kb) [file 40170_2018_174_MOESM4_ESM.pdf]

Supplementary Figure 3

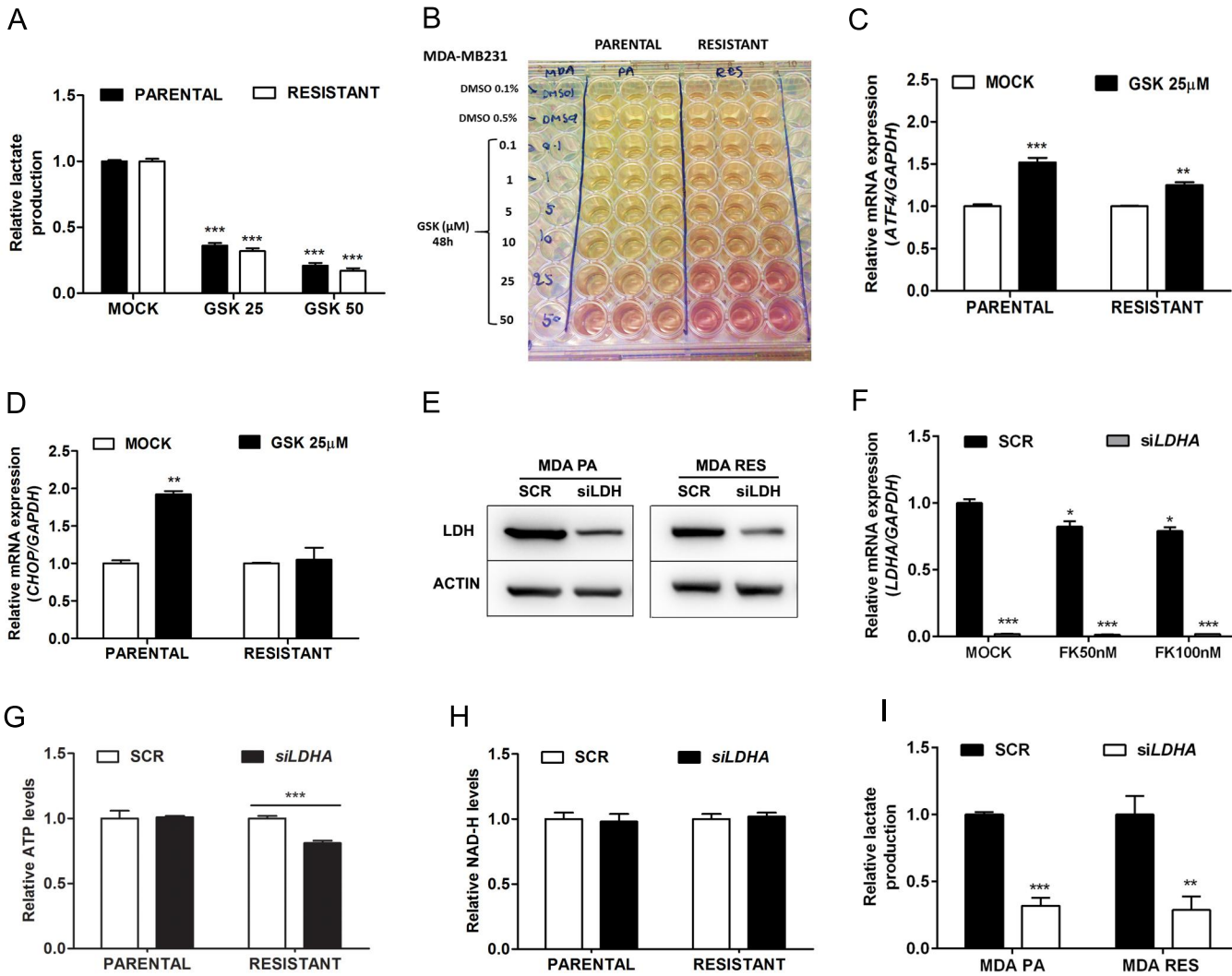

Supplement: Supplementary file 5 — Figure S3. A MDA PA and MDA RES cells were treated with 25 and 50 μM GSK for 48 h. Lactate production was determined. B Reduction of acidity was observed in GSK treatments. MDA PA and MDA RES cells were treated with various concentrations of GSK (0.1–50 μM) for 48 h. A decrease of acidity was visualized by changing of medium colors from red to yellow (basic to acidic). C, D Expression of ATF and CHOP mRNA along with 25 μM GSK treatment for 48 h in MDA. E Western blot showing downregulation of LDHA level in LDHA silencing MDA cells conducted by siRNA. ACTIN was used as a loading control. F FK866 decreased LDHA mRNA levels. MDA RES cells were transiently transfected with siLDHA. Twenty-four hours after silencing, cells were treated with 50 and 100 nM FK866 for 48 h. Expression of LDHA was detected. G–I MDA PA and MDA RES cells were transiently transfected with siLDHA. Forty-eight hours post transfection, ATP (G), NAD(H) (H), and lactate production (I) were measured (*p < 0.05, **p < 0.01, ***p < 0.001). (PDF 2727 kb) [file 40170_2018_174_MOESM5_ESM.pdf]
